# Single level versus multi-level lumbar interbody fusion for lumbar degenerative diseases: a systematic review and meta analysis

**DOI:** 10.1186/s13018-026-06778-4

**Published:** 2026-03-14

**Authors:** Dana Ibrahim Alharbi, Waleed Osama Samarkandi, Leen Saleh Albraik, Layan Saleh Albraik, Muteb Nasser Alotaibi, Nasser Ahmad Alsaleh, Usman Salman Ali, Yaser Ali Alnafesh, Abdullah Hassan Latifah, Abdulrahman Alnwiji, Mohammed Omar Alamodi, Raed A. Albar, Ayman MA Mohamed

**Affiliations:** 1 College of Medicine, Alfaisal University, 11533 Riyadh, Saudi Arabia; 2 College of Medicine, AlMaarefa University, P.O. Box 71666, 11597 Riyadh, Saudi Arabia

**Keywords:** Lumbar fusion, LIF, Spondylolisthesis, Single level

## Abstract

**Background:**

Degenerative spondylolisthesis affects approximately 39 million patients worldwide. While consensus supports decompression with fusion for single-level pathology, optimal surgical approaches for multi-level disease remain disputed. Despite the frequency of this clinical presentation, evidence comparing outcomes between Single-Level versus Multi-Level interbody fusion procedures is surprisingly scarce. This study aims to determine how the level of interbody fusion extent impacts outcomes in patients undergoing lumbar fusion for degenerative spondylolisthesis.

**Methods:**

Our systematic review methodology involved comprehensive database searches (Web of Science, Scopus, PubMed, and Cochrane Library) from inception through April 2025. Two independent reviewers performed article screening, data extraction, and quality assessment. Statistical analyses used R software (v4.4.2), with outcomes reported as risk ratios for categorical variables and mean differences for continuous measures (95% CI). The certainty of evidence was assessed using the GRADE approach.

**Results:**

Our meta-analysis evaluated 10 studies (*N* = 1430 patients; 971 Single-Level, 366 Double-Level, 198 Multi-Level fusions). Single-Level procedures demonstrated 41% lower revision rates (RR = 0.59 [0.40–0.86], *p* = 0.007). Operative advantages included reduced surgical time (−60.73 min [− 80.89 to − 40.57], *p* < 0.001), blood loss (-286.99mL [− 496.71 to − 77.27], *p* = 0.007), and hospitalization (−1.22 days [− 2.09 to − 0.34], *p* = 0.006). Oswestry Disability Index (ODI) scores showed borderline improvement (-3.90 [− 7.89 to 0.10], *p* = 0.06). Screw loosening decreased by 84% (RR = 0.16 [0.08–0.34], *p* < 0.001). We observed no significant differences in lumbar lordosis (-0.01 [− 1.75 to 1.72], *p* = 0.99), infection rates (RR = 0.49 [0.19–1.25], *p* = 0.13), adjacent segment deterioration, vascular injuries, or dural tears. The certainty of evidence ranged from low to very low, and high heterogeneity was observed in perioperative outcomes.

**Conclusions:**

Single-level fusion may offer a more favorable perioperative profile than double- or multi-level constructs, including lower revision risk, shorter operative time, reduced blood loss, shorter hospitalization, and fewer screw loosening events in pooled analyses. However, complications did not differ significantly between groups. Given substantial heterogeneity for perioperative outcomes and generally low to very low certainty of evidence, these findings should be interpreted cautiously and individualized to patient pathology and surgical context.

**Supplementary Information:**

The online version contains supplementary material available at 10.1186/s13018-026-06778-4.

## Introduction

Degenerative spondylolisthesis (DS) affects approximately 39 million individuals globally, with posterior decompression and lumbar arthrodesis representing standard interventional approaches for this pathology [1]. Management of multi-level DS disease presents a therapeutic dilemma, with divergent surgical philosophies evident in contemporary practice. Some surgeons advocate extensive fusion across all decompressed segments, citing concerns regarding iatrogenic instability following laminectomy, while others preferentially employ more limited fusion constructs despite performing multi-level decompressive procedures [2, 3]. This variation in surgical decision-making reflects the ongoing uncertainty regarding optimal treatment strategies for this complex patient population.

Previous literature established the superiority of combined decompression-fusion over isolated decompression for symptomatic single-level degenerative spondylolisthesis, demonstrating improved clinical outcomes [4, 5]. While single-level fusion efficacy is well-documented, comparative evidence regarding management strategies for multi-level stenosis with single-level spondylolisthesis remains limited [4, 5]. The Spine Patient Outcomes Research Trial (SPORT) investigation highlighted the prevalence of this clinical scenario, with 35% of degenerative spondylolisthesis patients demonstrating multi-level moderate-to-severe stenosis, and 57% of surgical patients undergoing multi-level decompression [6, 7].

Current surgical approaches vary considerably. Some surgeons restrict fusion to the demonstrably unstable segment while performing isolated decompression at additional stenotic levels. Others advocate multi-level fusion as prophylaxis against adjacent segment deterioration. Multiple investigations have demonstrated that isolated laminectomy increases segmental instability without concomitant fusion, suggesting that decompression above a fused segment may accelerate adjacent segment pathology through increased biomechanical stress [2, 8].

However, extended fusion constructs introduce additional considerations. Multi-level fusion procedures typically involve longer operative duration with potentially increased morbidity. Furthermore, extended constructs may accelerate adjacent segment deterioration through increased biomechanical demands at remaining mobile segments [9–13].

While comparative outcomes between single-level and multi-level fusion for multi-level stenosis have been documented in the literature, demonstrating limited utility for extending fusion beyond the spondylolisthetic level, the comparative efficacy of these approaches specifically for multi-level degenerative spondylolisthesis remains inadequately characterized [3, 14]. Notably, no previous meta-analyses have addressed this clinical question, with existing evidence limited to small observational studies. Consequently, our investigation aimed to determine the impact of level of interbody fusion extent on clinical outcomes in patients undergoing lumbar interbody fusion for degenerative spondylolisthesis.

## Materials and methods

Our methodology strictly followed established systematic review principles, implementing both Cochrane Handbook methodological standards and PRISMA reporting guidelines. The review was registered in OSF (Registration DOI: 10.17605/OSF.IO/WKQ5U) and adhered to the published protocol [15, 16].

### Literature search

A comprehensive literature search was implemented across four major bibliographic repositories (PubMed, Scopus, Web of Science, and Cochrane CENTRAL) from their establishment through April 2025. To ensure exhaustive literature capture, we augmented algorithmic database searching with manual citation tracking from qualifying studies and relevant systematic reviews. Search parameters incorporated precisely defined terminology combinations as documented in Table A1.

### Eligibility criteria

Study eligibility was assessed independently by two reviewers using predefined inclusion criteria, and studies were considered eligible if they met the following requirements: (1) enrolled adult participants (≥ 18 years) diagnosed with degenerative lumbar disease; (2) directly compared single-level fusion (two adjacent vertebrae) with double/multi-level fusion (three or more vertebrae); (3) were original research published in peer-reviewed, English-language journals with full-text availability; (4) included primary surgeries only (revision procedures excluded); (5) used standard instrumentation (pedicle screw/rod systems or standalone cages); (6) reported a minimum follow-up of at least 6 months; (7) reported at least one clinical outcome (e.g., ODI) and/or radiological outcome (e.g., fusion rate or adjacent segment disease); and (8) provided a complete list of excluded full-text articles with reasons in Supplementary Appendix C.

### Data collection

Data extraction was performed using a standardized collection sheet. We systematically recorded comprehensive information for each included study: unique identifier, intervention groups, methodological design, cohort size, follow-up duration, demographic characteristics (mean age with standard deviation, gender distribution, and mean BMI), clinical variables (tobacco usage rates, diabetes prevalence), disease-specific metrics to measure functional disability in patients with low back pain as (mean Oswestry Disability Index with standard deviation), diagnostic classification, pathology level, eligibility criteria, and principal conclusions.

#### Outcome definitions

To ensure consistency across studies, outcomes were defined a priori as follows: (1) Adjacent Segment Disease (ASD) was accepted as defined by each study, with documentation of whether ASD was radiographic (e.g., change in Pfirrmann grade) or symptomatic (e.g., requiring reoperation); (2) infection was defined as either deep or superficial surgical-site infection requiring clinical intervention; and (3) for the Oswestry Disability Index (ODI), we extracted the final follow-up scores as reported in each study. For analytical purposes, Single-Level refers to fusion at one motion segment, Double-Level refers to fusion involving two motion segments, and Multi-Level refers to fusion involving three or more motion segments. These definitions were applied consistently across study classification, subgroup analyses, and outcome synthesis .

### Quality assessment

Risk of bias for non-randomized comparative studies was assessed using the ROBINS-I tool, evaluating bias across domains of confounding, selection of participants, classification of interventions, deviations from intended interventions, missing data, measurement of outcomes, and selection of the reported result. Each study was judged as having low, moderate, serious, or critical risk of bias in each domain and overall [17]. The randomized controlled trial was assessed separately using the RoB-2 tool according to the Cochrane guidelines. Disagreements were resolved by discussion and consensus, and results are summarized in the risk-of-bias figures and Supplementary material [18].

### Certainty of evidence (GRADE)

The certainty of evidence for each key outcome (including revision surgery, ODI, operative time, blood loss, length of stay, infection, screw loosening, and ASD) was evaluated using the GRADE approach. For each outcome, we considered risk of bias, inconsistency, indirectness, imprecision, and publication bias, downgrading or upgrading the certainty as appropriate. Randomized evidence started as high certainty and observational evidence as low certainty, with subsequent rating adjustments based on these domains. Final ratings (high, moderate, low, or very low certainty) are presented in a Summary of Findings table in the Supplementary material [19].

### Data synthesis

All statistical analyses were performed using R version 4.4.2. For continuous outcomes, we calculated pooled mean differences (MD), while dichotomous variables were analyzed using risk ratios (RR), each with corresponding 95% confidence intervals. Assessment of inter-study heterogeneity employed dual methodological approaches: I² statistics (with values ≥ 50% denoting substantial heterogeneity) and chi-square testing (*p* < 0.10 indicating significant heterogeneity). Throughout our analyses, we adopted the standard significance threshold of *p* < 0.05 for statistical inference. Because fewer than 10 studies were available for most outcomes, formal publication-bias assessments (e.g., funnel plot asymmetry tests) and meta-regression were not performed, consistent with common methodological guidance [15, 16].

## Results

### Literature search results

**Fig. 1:**
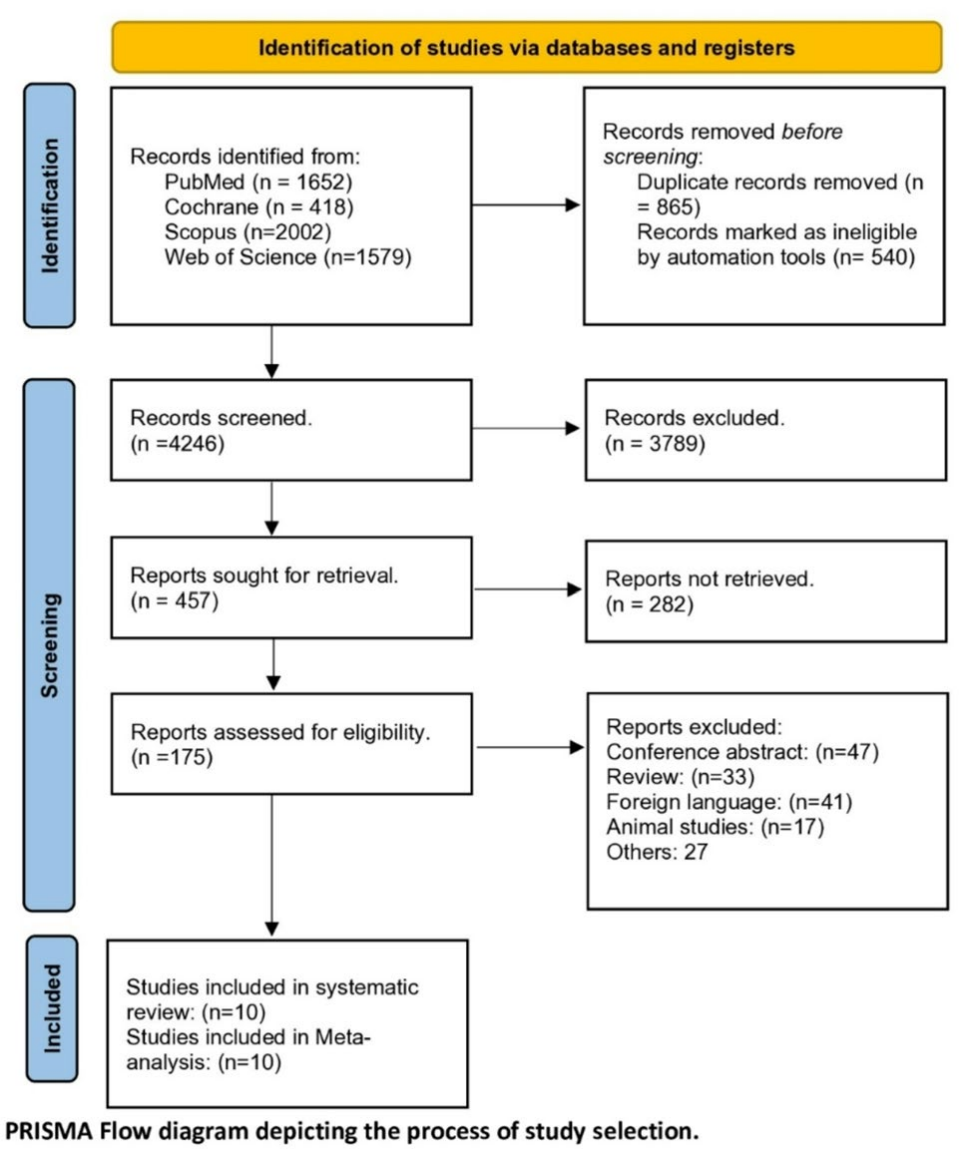
PRISMA flow diagram for studies selection process

We performed an exhaustive systematic search across several electronic databases, yielding 5651 initial citations. Pre-screening filtration eliminated 2607 records (865 duplicates and 540 automation-flagged ineligible entries). The remaining 4246 citations underwent preliminary assessment, with 4071 subsequently excluded during title and abstract review. Upon comprehensive evaluation of 175 full-text manuscripts, 138 failed to satisfy inclusion parameters. Ten studies ultimately fulfilled all eligibility criteria and were incorporated into both our qualitative synthesis and quantitative meta-analysis [2, 20–28]. The stepwise selection methodology adhered to PRISMA guidelines and is visually represented in Fig. 1.

### Included studies characteristics

Our meta-analysis included 10 studies (nine observational, and one prospective randomized trial), encompassing 1,430 patients who underwent lumbar spinal fusion procedures (971 single-level, 366 double-level, and 198 multi-level fusions). Mean patient age ranged from 35.7 to 70.1 years with variable gender distribution (18.7–66.7% male) and BMI values (23.3–29.1 kg/m²). The surgical techniques included posterior lumbar interbody fusion (PLIF), anterior lumbar interbody fusion (ALIF), or lateral lumbar interbody fusion (LLIF), primarily addressing degenerative conditions such as spondylolisthesis, spinal stenosis, degenerative disc disease, and scoliosis, most commonly at L4-L5 and L5-S1 levels. Important considerations for patient selection emerged, particularly regarding osteoporosis, sagittal balance parameters, and adjacent segment disease risk, with some studies specifically recommending enhanced fixation techniques for high-risk patients or hybrid approaches for multi-level pathologies. Baseline Characteristics and Summary of the included studies are shown in Table 1.

**Table 1:** Baseline characteristics and Summary of the included studies

Study ID	Arms	Study Design	Sample Size	Follow Up	Age (Years), Mean (Sd)	Sex (Male), *N* (%)	Body Mass Index (Kg/M2), Mean (Sd)	Tobacco Use, *N* (%)	Diabetes, *N* (%)	ODI, MEAN (SD)	Diagnosis	Level of Disease	Inclusion Criteria	Conclusions
Guo 2020	Single level	Retrospective study	100	31.8 m	64.2 (9.6)	19 (19)	23.5 (3)	NA	NA	52.2 (8.9)	**Degenerative lumbar diseases**,** including**: *Lumbar spinal stenosis: 137 patients. *Lumbar spondylolisthesis: 77 patients. *Degenerative scoliosis: 3 patients.	L4-L5	(1) patients diagnosed with degenerative lumbosacral disease (2) patients whose lumbar vertebral BMD measured by dual-energy X-ray absorptiometry less than or equal to -2.5 SD; and (3) patients underwent posterior lumbar fusion (posterior lumbar interbody fusion or transforaminal lumbar interbody fusion) combined with pedicle screw fixation.	Owing to the high rate of screw loosening in cranial and caudal vertebra, osteoporotic patients with double-level or multi-level pedicle screw fixation benefited less than those with single-level pedicle screw fixation. Larger PI-LL, larger PT, and lumbosacral fixation are other risk factors for the loosening of screw. An instrument with stronger holding strength at cranial and caudal pedicle screws is recommended for those high-risk patients.
	Double-level		73		64.9 (9.8)	13 (18.7)	23.3 (3)	NA	NA	51.7 (8.1)		L4-L5 and L5-S1		
	Multi-level		44		67 (7.6)	5 (11.4)	24.4 (2.7)	NA	NA	53.3 (7.5)		L2-L5		
Hiyama 2025	Single-level Lateral Lumbar Interbody Fusion (LLIF)	Retrospective study	89	12 m	70.1 (11.1)	55 (61.7)	24.3 (3.6)	21 (24)	NA	NA	**Lumbar degenerative disease (LDD)**,** including**: 1-Lumbar canal stenosis with degenerative spondylolisthesis: 112 patients (81%) 2-Degenerative lumbar scoliosis: 11 patients (8%) 3-Foraminal stenosis: 7 patients (5%) 4-Lumbar disc herniation: 7 patients (5%) 5-Synovial cysts: 2 patients (1%)	L4-L5 (73%), L3-L4 (22%), L2-L3 (3%), L1-L2 (1%)	Adults aged 18 years and older with lumbar degenerative disease (LDD), Persistent low back pain, leg pain, or numbness unrelieved by at least three months of conservative treatment, and Patients who underwent LLIF combined with posterior fixation between May 2018 and January 2023.	Short-term clinical outcomes suggested that both single-level and multi-level LLIF effectively improved pain and QOL outcomes in patients with LDD
	Multi-level LLIF		50		69.8 (8.4)	29 (58)	24.7 (3.2)	9 (18)	NA	NA		L3-L4 (46%), L4-L5 (37%), L2-L3 (15%), L1-L2 (2%).		
Levin 2007	Single level	Retrospective study	9	32 m	35.67 (20.9)	6 (66.7)	26.9	NA	NA	NA	Discogenic low back pain associated with degenerative disc disease (DDD) in the lumbosacral spine (L3–S1).	L3–S1	Age 18–60 years, At least 6 months of failed nonoperative therapy, DDD at one or two adjacent vertebral levels between L3 and S1, confirmed by radiographic evidence (CT, MRI, discography, plain film, myelography, or flexion-extension films), Oswestry Low Back Pain Disability Questionnaire score of at least 20/50 (40%).	Patients undergoing 1- and 2-level ProDisc total disc replacement spent significantly less time in the operating room (OR) and had less estimated blood loss (EBL) than the control group (circumferential fusion). Charges were significantly lower for total disc replacement (TDR) compared with circumferential fusions in the 1-level patient group, while charges were similar in the 2-level group.
	Double-level		8	29 m	38 (12.5)	5 (62.5)	26.7	NA	NA	NA				
Li 2018	Single level	Prospective Randomized Control Study	50	48 m	53.2 (7.2)	27 (57.4)	24.4 (5.7)	14 (29.8)	NA	44.3 (16.4)	Multi-level lumbar degenerative disease (LDD) neurogenic claudication or radicular leg pain with associated neurologic signs	L4–L5 **or** L5–S1	(1) neurogenic claudication or radicular leg pain with associated neurologic signs, (2) preoperative radiologic examination showing multi-level (consecutive 3-level or 4-level) LDD, and (3) no response toatleast 6 months of conservative treatment.	A hybrid technique including 1-level interbody fusion and multi-level posterolateral fusion is recommended for patients with multi-level LDD.
	Multi-level		59		51.8 (6.8)	24 (52.2)	26 (6.3)	12 (26.1)	NA	48.7 (18.2)		NA		
Martini 2020	Single level	Retrospective study	268	42 m	52.6 (0.8)	123(45.9)	29 (11.45)	NA	NA	NA	Degenerative disc disorders	NA	all cases involving 1-level or 2-level ALIF procedures performed for degenerative disc disorders between 2008 and 2016.	Our findings support a biomechanical hypothesis of ASD onset after fusion, suggesting that the risk of ASD after ALIF lies primarily in the number of levels fused rather than any demographic or intraoperative variables.
	Double-level		136		53.6 (1.2)	52(38.2)	28.1 (5.8)	NA	NA	NA		NA		
Sakaura 2013	Single level	Retrospective study	92	40 m	65 (33.14)	40 (43.5)	NA	NA	NA	NA	Degenerative lumbar spondylolisthesis (DS)	L3–L5 (17 patients), L2–L4 (1 patient), L4–L6 (1 patient), L4–S1 (1 patient).	All patients with lumbar DS have been treated using PLIF with pedicle screws in our hospital since 2005 irrespective of severity of slippage, patient age, and bone quality. Based on this surgical strategy, 21 consecutive patients with 2-level DS underwent 2-level PLIF between March 2005 and July 2008.	The clinical outcome of 2-level PLIF for 2-level lumbar DS was satisfactory, although surgery related complications including symptomatic adjacent-segment disease were not negligible.
	Double-level		20	35.4 m	63.7 (27.9)	6 (30)	NA	NA	NA	NA		L4–L5 (82 patients), L3–L4 (7 patients), L5–S1 (2 patients), L5–L6 (1 patient)		
Singh 2025	Single level	Retrospective cohort study	111	27 m	51.76 (10.65)	48 (43.2)	29 (5.67)	NA	NA	NA	Degenerative spine pathology	Fusion at L4-L5 or L5-S1	Adult patients (≥ 18 years) with degenerative spine pathology, Elective ALIF surgery using a PEEK or titanium cage, True anterior retroperitoneal approach with the assistance of a general surgeon.	higher procedural time, length of stay, and approach-related complications than one-level ALIF. Although there were minor improvements in alignment with two-level ALIF, PROMs were comparable with improvements from baseline to last follow-up.
	Double-level		47		50.70 (11.55)	20 (42.6)	28.1 (5.16)	NA	NA	NA		Fusion at L4-S1		
Smorgick 2014	Single level	Subanalysis of a multicenter randomized and observational study (SPORT - Spine Patient Outcomes Research Trial)	130	48 m	66.7 (10.3)	49 (37.7)	29.1 (7.2)	10 (8)	17 (13)	43.3 (16.6)	Multi-level lumbar stenosis and single-level degenerative spondylolisthesis (DS)	NA	Patients with neurogenic claudication or radicular leg pain with associated neurologic signs, Spinal stenosis shown on cross-sectional imaging, Single-level degenerative spondylolisthesis (DS) shown on standing lateral radiographs, Persistent symptoms for at least 12 weeks.	Decompression and single level fusion and decompression and multi level fusion provide similar outcomes in patients with multi-level lumbar stenosis and single level degenerative spondylolisthesis.
	Multi-level		77		66.5 (9)	26 (33.7)	28.6 (5.7)	6 (8)	9 (12)	45.7 (17)				
Barrett-Tuck 2017	Single level	Retrospective study	18	12 m	57.7 (39.3)	12 (48)	NA	NA	7 (28)	NA	Degenerative spinal conditions (e.g., disc degeneration, instability, stenosis, disc rupture/herniation, spondylolisthesis, facet disease) who failed conservative management	L3-L4: 8 patients (44%), L4-L5: 8 patients (44%), and L5-S1: 2 patients (11%).	Patients with persistent low back pain and/or recurrent disc herniation refractory to at least 6 months of conservative care, Diagnosed with degenerative spinal conditions (e.g., disc degeneration, instability, stenosis, disc rupture/herniation, spondylolisthesis, facet disease).	the VariLift^®^ device used in single or two-level PLIF provided effective symptom relief and produced a high fusion rate without the need for supplemental fixation
	Double-level		7									L2-L4: 1 patient (14%), L3-L5: 1 patient (14%), L4-S1: 5 patients (71%)		
Harada 2021	Single level	Retrospective cohort study	203	6 m	64.78 (10.57)	73 (35.9)	NA	23 (11.5)	23 (11.33)	NA	Low-grade degenerative spondylolisthesis (Grade I or II) with lumbar radiculopathy and/or neurogenic claudication after failure of conservative treatment	NA	Patients undergoing primary instrumented posterolateral lumbar spinal fusion for low-grade spondylolisthesis (Grade I or II), Patients with lumbar radiculopathy and/or neurogenic claudication after failure of conservative treatment.	Patients in multi-level fusions experienced less improvement in back pain, had more complications, and were more commonly discharged to a facility compared with single-level PLF patients. These findings are important for operative planning, for setting appropriate preoperative expectations, and for risk stratification in patients undergoing posterior lumbar fusion for low grade spondylolisthesis.
	Double-level		95		64.87 (8.77)	30 (31.5)	NA	9 (9.68)	11 (11.58)	NA		NA		
	Multi-level		18		69.22 (8.59)	5 (27.8)	NA	2 (11.11)	1 (5.56)	NA		NA		

ALIF: Anterior Lumbar Interbody Fusion; ASD: Adjacent Segment Disease; BMI: Body Mass Index; CT: Computed Tomography; DDD: Degenerative Disc Disease; DS: Degenerative Spondylolisthesis; EBL: Estimated Blood Loss; LLIF: Lateral Lumbar Interbody Fusion; LDD: Lumbar Degenerative Disease; MRI: Magnetic Resonance Imaging; NA: Not Available; ODI: Oswestry Disability Index; OR: Operating Room; PI–LL: Pelvic Incidence Minus Lumbar Lordosis; PLF: Posterolateral Fusion; PLIF: Posterior Lumbar Interbody Fusion; PROMs: Patient-Reported Outcome Measures; PT: Pelvic Tilt; PEEK: Polyetheretherketone; QOL: Quality of Life; SD: Standard Deviation; SPORT: Spine Patient Outcomes Research Trial; TDR: Total Disc Replacement

### Quality assessment

#### Risk of bias assessment

On ROBINS-I evaluation, the majority of studies were judged at moderate overall risk of bias, reflecting retrospective design with residual confounding and incompletely characterized loss to follow-up, whereas a few small, highly selected series were rated at serious risk of bias due to pronounced selection issues, limited control of key prognostic factors, and poorly documented missing data. For risk of bias assessment using the ROB2 tool, the Li (2018) study demonstrated low risk of bias across all five domains: randomization process, deviations from intended interventions, missing outcome data, measurement of the outcome, and selection of the reported result (Table A2 and Figure 9).

#### Certainty assessment

In GRADE assessment, the certainty of evidence was low for revision surgery and screw loosening and very low for all other outcomes. Single-level fusion was associated with lower risks of revision (RR 0.59, 95% CI 0.40–0.86) and screw loosening (RR 0.16, 95% CI 0.08–0.34), and with shorter operative time, less blood loss, and reduced length of stay, but these estimates were downgraded for serious risk of bias, extreme heterogeneity, and (for most outcomes) imprecision and indirectness. For ODI, incision infection, and adjacent segment deterioration, wide confidence intervals crossing the null and variability in definitions further reduced certainty, so these effects should be interpreted cautiously despite the consistent directional trend favoring single-level construct. Supplementary Table 1.

### Outcomes

#### Revision surgery

**Fig. 2:**
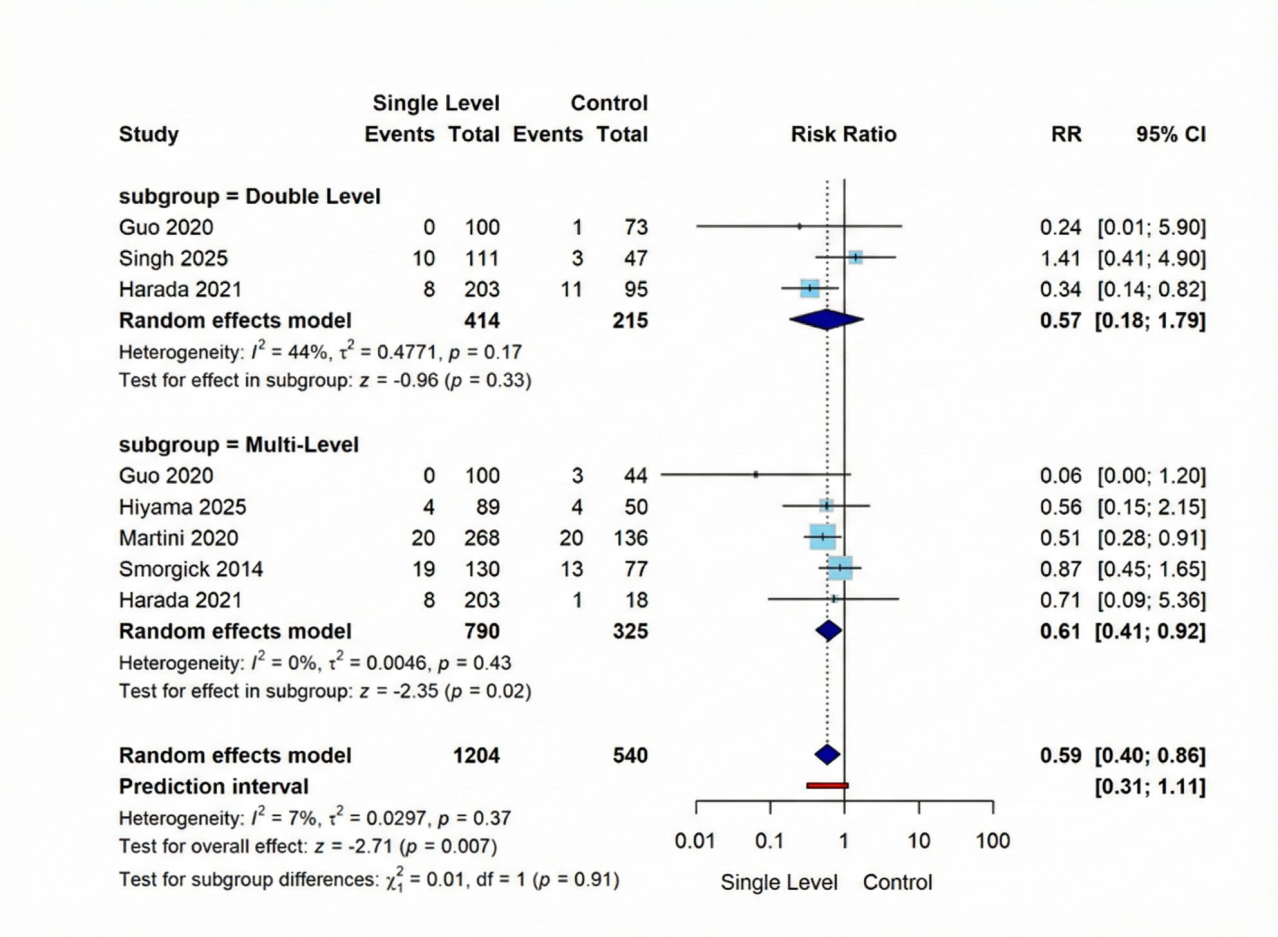
Forest plot for random effect model meta-analysis for revision surgery outcome

Our overall pooled analysis showed that Single-Level interbody fusion demonstrated a statistically significant 41% reduction in events compared to Double and Multi-Level groups collectively (RR = 0.59, 95% CI [0.40; 0.86], *p* = 0.007). Subgroup analysis revealed distinct patterns: the Double-Level subgroup (three studies, 414 Single-Level vs. 215 Multi-Level Group participants) showed a non-significant difference (RR = 0.57, 95% CI [0.18; 1.79], *p* = 0.33) ; while the Multi-Level subgroup (5 studies, 790 Single-Level vs. 325 Multi-Level Group participants) demonstrated a significant 39% reduction in events, favoring Single-Level (RR = 0.61, 95% CI [0.41; 0.92], *p* = 0.02). Overall analysis showed no heterogeneity across all studies (I² = 7%, *p* = 0.37). Figure 2.

#### Change in lumbar lordosis

**Fig. 3:**
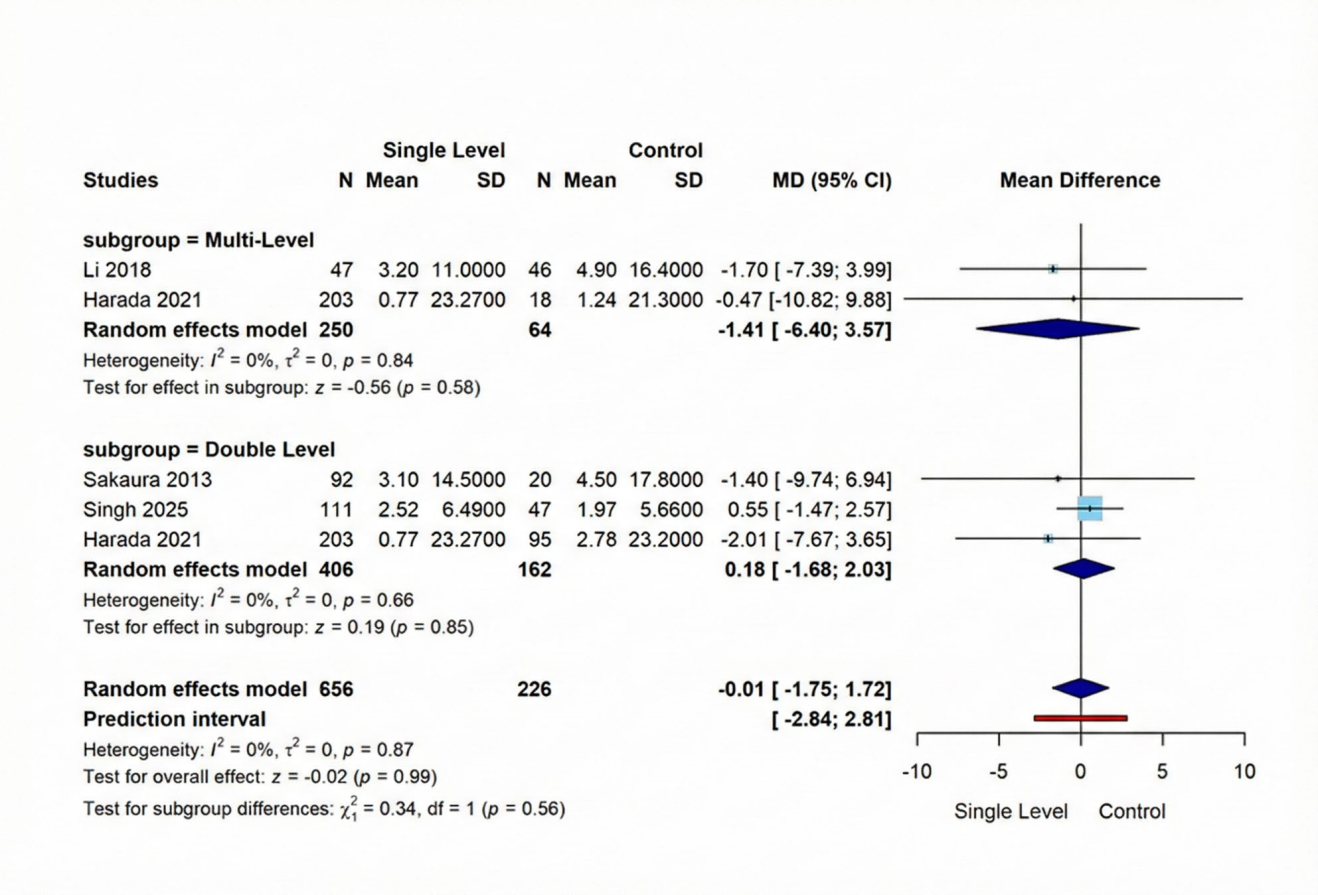
Forest plot for random effect model meta-analysis for change in lumbar lordosis

In this outcome, our meta-analysis comprised five studies comparing Single-Level versus Comparison Group interventions showed no significant difference between interventions (MD = −0.01, 95% CI [−1.75; 1.72], *p* = 0.99) with homogeneity across studies (I² = 0%, *p* = 0.87). Subgroup analysis revealed consistent findings: the Multi-Level subgroup (2 studies, 250 vs. 64 participants) showed a non-significant difference (MD = −1.41, 95% CI [−6.40; 3.57], *p* = 0.58), also the Double-Level subgroup (3 studies, 406 vs. 162 participants) demonstrated a non-significant difference (MD = 0.18, 95% CI [-1.68; 2.03], *p* = 0.85). Figure 3 shows these results visually.

#### Oswestry disability index (ODI)

**Fig. 4:**
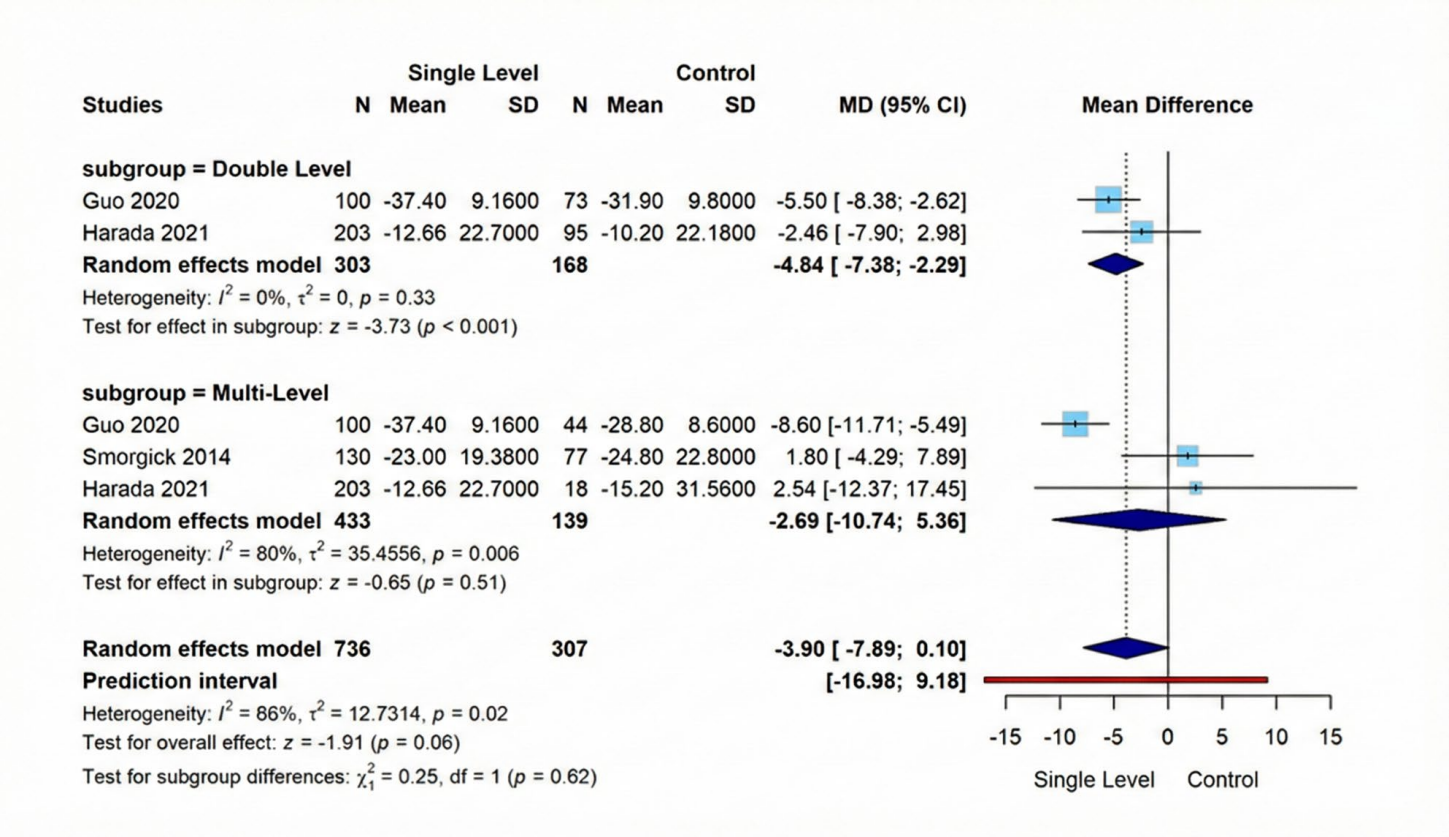
Forest plot for random effect model meta-analysis for Oswestry Disability Index outcome

Our meta-analysis evaluating revealed a marginally non-significant mean difference of −3.90 (95% CI: −7.89 to 0.10, *p* = 0.06), with considerable inter-study variability (I²=66%, *p* = 0.02). Further stratification demonstrated that the Double-Level subgroup exhibited significant benefits from Single-Level intervention (MD= −4.84, 95% CI: −7.38 to −2.29, *p* < 0.001) with homogeneous findings across studies (I²=0%, *p* = 0.33). In contrast, analysis of the Multi-Level subgroup (3 studies, *n* = 572) yielded inconclusive results (MD= −2.69, 95% CI: −10.74 to 5.36, *p* = 0.51). Figure 4 displays these results visually.

#### Operation time

**Fig. 5:**
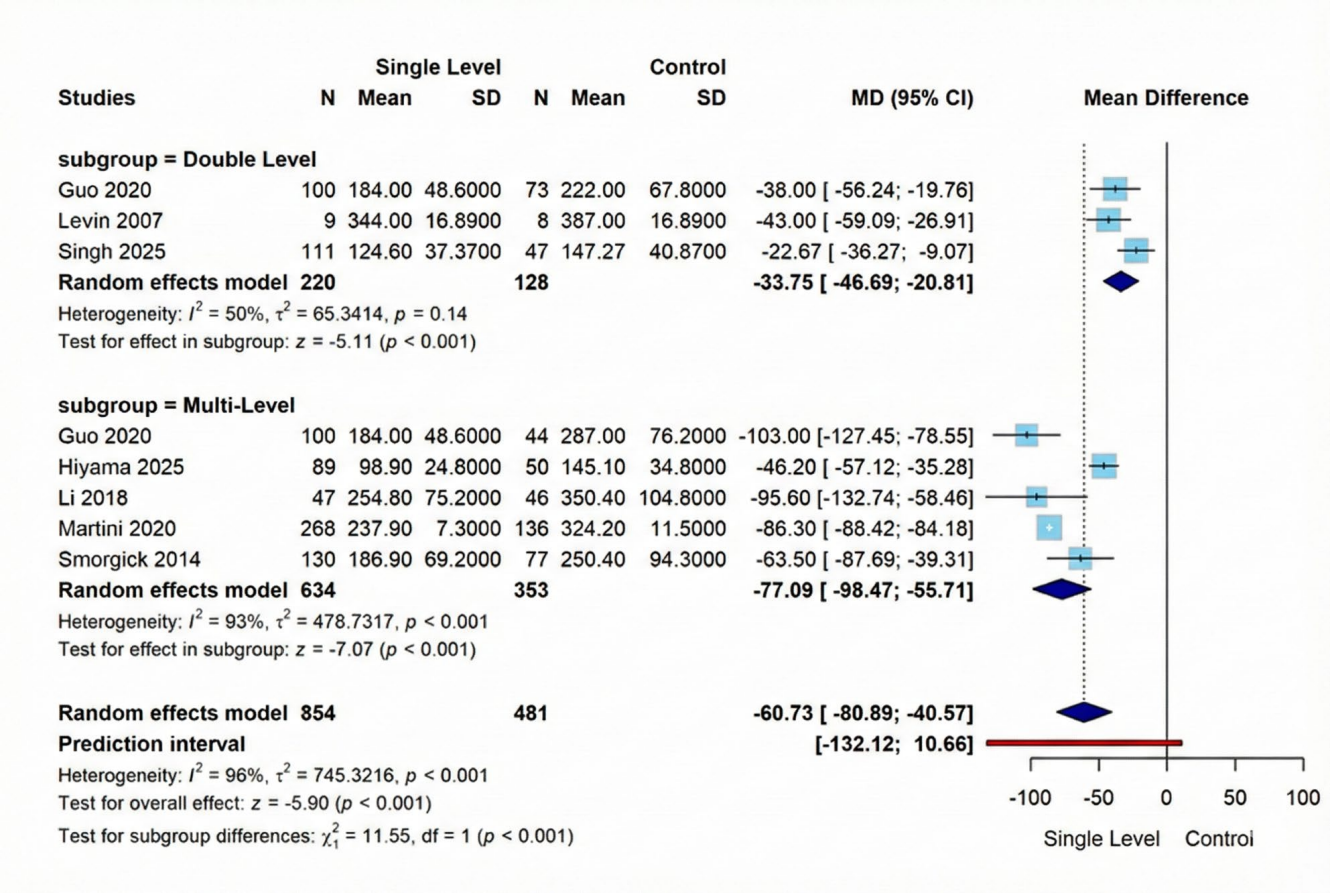
Forest plot for random effect model meta-analysis for operation time outcome

As to operation time, our pooled analysis of seven studies, we found a statistically significant mean difference of -60.73 (95% CI [-80.89; -40.57], *p* < 0.001) favoring Single-Level intervention, albeit with considerable heterogeneity (I² = 96%, *p* < 0.001). The Double-Level subgroup (3 studies, 220 vs. 128 participants) demonstrated a significant effect favoring Single-Level (MD = −33.75, 95% CI [−46.69; −20.81], *p* < 0.001) with moderate heterogeneity (I² = 50%, *p* = 0.14), while the Multi-Level subgroup (5 studies, 634 vs. 353 participants) showed a stronger effect (MD = −77.09, 95% CI [−98.47; −55.71], *p* < 0.001) with substantial heterogeneity (I² = 93%, τ² = 478.7317, *p* < 0.001). Figure 5 illustrates these results visually.

#### Blood loss

**Fig. 6:**
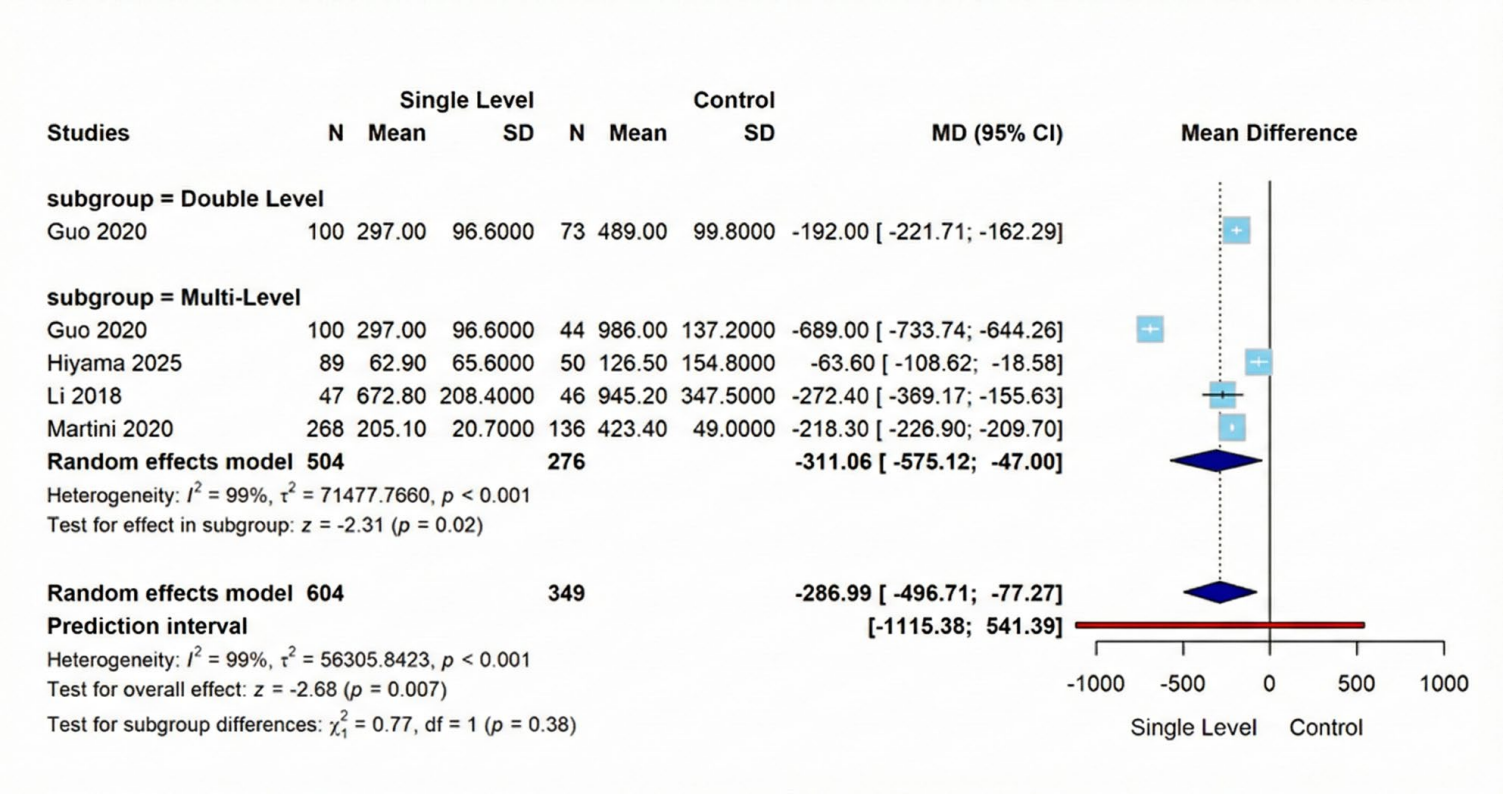
Forest plot for random effect model meta-analysis for blood loss outcome

This meta-analysis comparing Single-Level versus Comparison Group interventions across four studies demonstrated a statistically significant mean difference of −286.99 (95% CI [−496.71; −77.27], *p* = 0.007) favoring Single-Level intervention, albeit with extreme heterogeneity (I² = 99%, τ² = 56305.8423, *p* < 0.001). Similarly, the Multi-Level subgroup (4 studies, 504 vs. 276 participants) demonstrated a significant effect favoring Single-Level (MD = −311.06, 95% CI [−575.12; −47.00], *p* = 0.02), with substantial heterogeneity (I² = 99%, τ² = 71477.7660, *p* < 0.001). Figure 6 shows these results visually.

#### Length of hospital stay

**Fig. 7:**
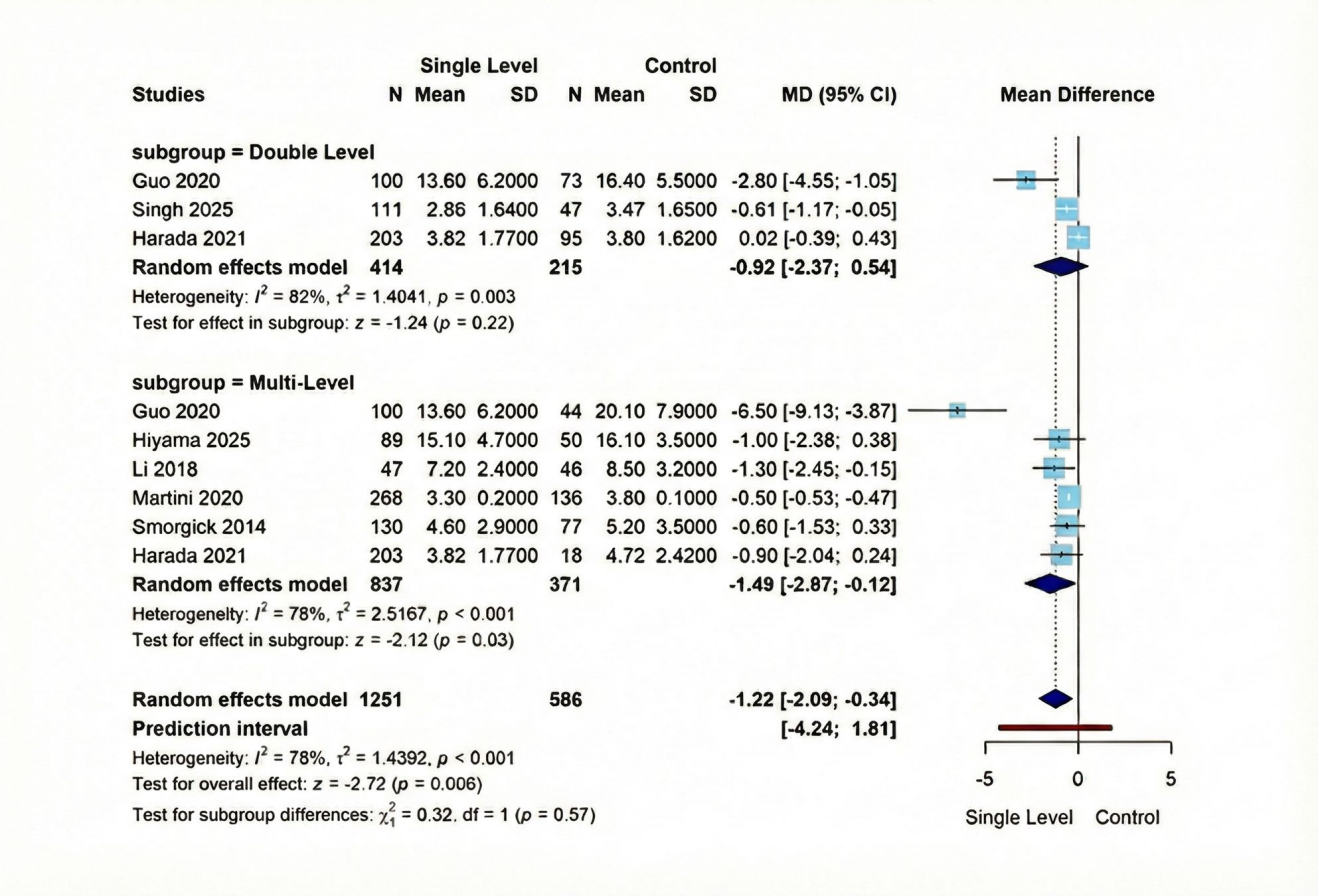
Forest plot for random effect model meta-analysis for length of hospital stay outcome

Regarding this outcome, our meta-analysis comprising seven studies demonstrated a statistically significant mean difference of −1.22 (95% CI [−2.09; −0.34], *p* = 0.006) favoring Single-Level intervention, with substantial heterogeneity (I² = 78%, τ² = 1.4392, *p* < 0.001). Subgroup analysis revealed differential patterns: the Double-Level subgroup (3 studies, 414 vs. 215 participants) showed a non-significant effect (MD = −0.92, 95% CI [-2.37; 0.54], *p* = 0.22) with substantial heterogeneity (I² = 82%, τ² = 1.4041, *p* = 0.003), while the Multi-Level subgroup (6 studies, 837 vs. 371 participants) demonstrated a significant effect favoring Single-Level (MD = −1.49, 95% CI [−2.87; −0.12], *p* = 0.03), also with substantial heterogeneity (I² = 78%, τ² = 2.5167, *p* < 0.001). Figure 7 displays these results visually.

#### Incision infection

**Fig. 8:**
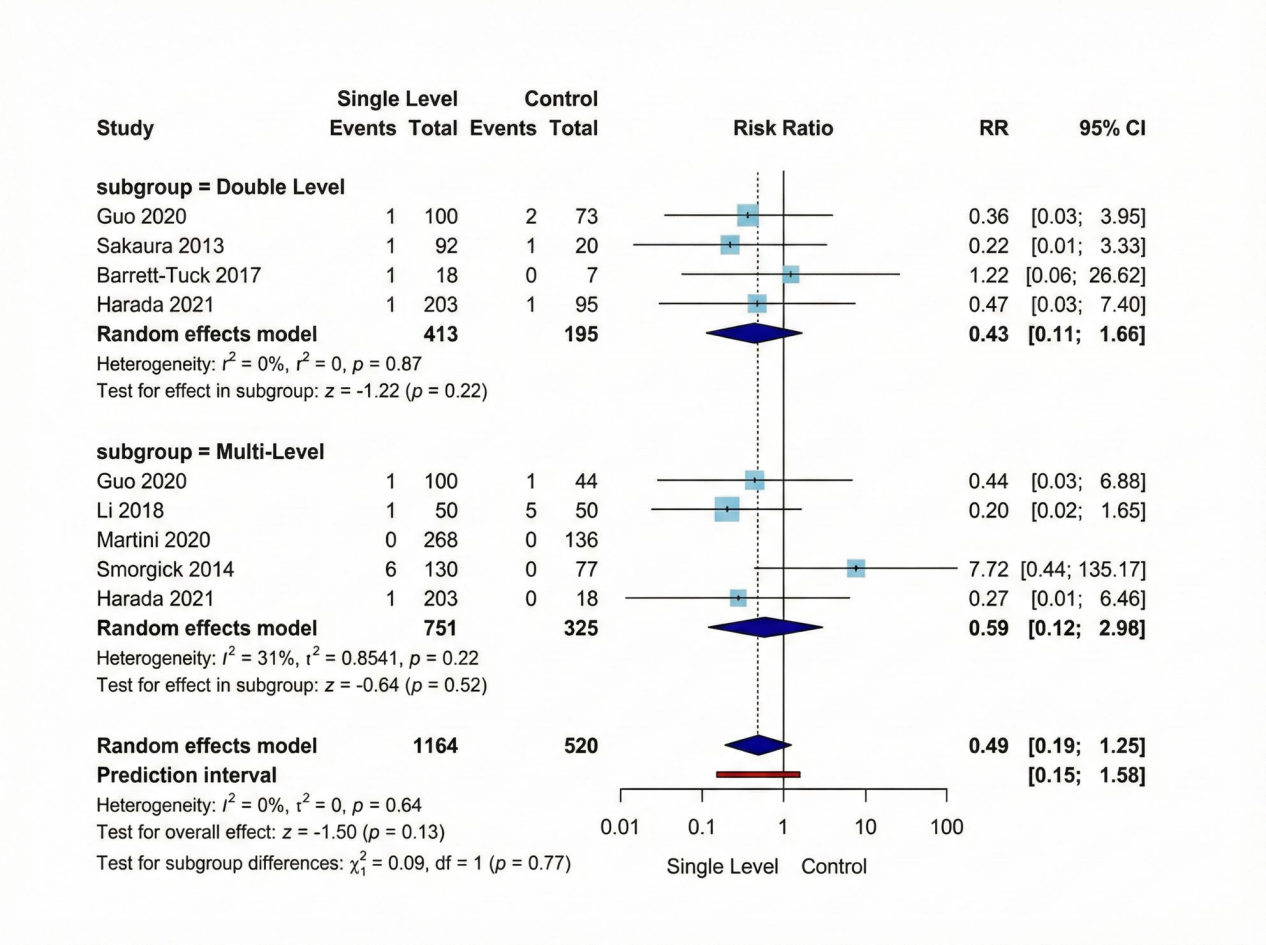
Forest plot for random effect model meta-analysis for incision infection outcome

For incision infection outcome, our meta-analysis comprising six studies did not show any significant difference between Single-Level and compared Multi-Level interventions (RR = 0.49, 95% CI [0.19; 1.25], *p* = 0.13). Similarly, subgroup analysis revealed that the Double-Level subgroup (4 studies, 413 Single-Level vs. 195 Multi-Level Group participants) showed a non-significant effect (RR = 0.43, 95% CI [0.11; 1.66], *p* = 0.22) with no heterogeneity (I² = 0%, τ² = 0). The Multi-Level subgroup (5 studies, 751 Single-Level vs. 325 Multi-Level Group participants) demonstrated also a non-significant effect (RR = 0.59, 95% CI [0.12; 2.96], *p* = 0.52). Overall heterogeneity across all studies was negligible (I² = 0%, *p* = 0.64). Figure 8 shows these results visually.

#### Sensitivity analysis

Leave-one-out sensitivity analyses showed that the pooled effects for perioperative outcomes were highly robust. For length of stay, operating time, blood loss, and ODI, omitting any single study produced very similar mean differences and confidence intervals, with all models consistently favoring the same treatment arm and remaining statistically significant. Although between-study heterogeneity remained high for length of stay, operating time, and blood loss, it did not materially diminish when any individual trial was removed, indicating that no single study was driving the overall effect. (Supplementary Figs. 1–4)

#### Secondary outcomes

Screw loosening, was substantially lower following Single-Level procedures versus Comparison Group interventions (RR = 0.16, 95% CI [0.08–0.34], *p* < 0.001). This represents a notable 84% reduction in risk across the three analyzed studies (combined *n* = 429), with findings demonstrating consistency (I² = 22%). Our evaluation of adjacent segment deterioration (ASD) across four comparative investigations (total *n* = 777) indicated no significant difference (RR = 0.62, 95% CI [0.28–1.35], *p* = 0.23), with remarkable consistency between studies (I² = 0%). Regarding vascular injuries, they exhibited a non-significant difference (RR = 0.28, 95% CI [0.06–1.35], *p* = 0.11). Similarly, dural tear occurrence showed statistically non-significant reduction with Single-Level approaches in our three-study analysis of 784 patients (RR = 0.55, 95% CI [0.26–1.16], *p* = 0.12**)** (Figures 10, 11, 12, 13).

## Discussion

Our meta-analysis of 10 studies (nine observational and one prospective randomized trial) encompassing 1430 patients undergoing lumbar spinal interbody fusion demonstrated several significant advantages for Single-Level procedures compared to Double and Multi-Level fusions. Most notably, Single-Level fusion was associated with a 41% reduction in revision surgery rates (*p* = 0.007), substantially shorter operation times (MD: −60.73 min, *p* < 0.001), significantly reduced intraoperative blood loss (mean difference: −286.99 ml, *p* = 0.007), and shorter hospital stays (MD: −1.22 days, *p* = 0.006). Additionally, we observed an 84% reduction in screw loosening complications with Single-Level procedures (*p* < 0.001). Functional outcomes measured by Oswestry Disability Index showed a trend favoring Single-Level fusion, particularly when compared to Double-Level procedures (*p* < 0.001). Interestingly, no significant differences were observed in lumbar lordosis changes, incision infection rates, adjacent segment deterioration, vascular injuries, or dural tear occurrence.

The notable distinction between single-level and multi-level LIF cohorts in perioperative parameters could be justified as follows. These findings align with the anticipated increased procedural complexity associated with addressing multiple vertebral segments. The extended surgical duration in multi-level interventions likely reflects the required additional technical steps, while heightened blood loss presumably results from more extensive tissue manipulation and exposure requirements. Furthermore, hospital length-of-stay was moderately increased in the multi-level cohort, suggesting potentially delayed rehabilitation initiation or extended recovery requirements following more comprehensive surgical intervention [23].

Our findings suggest that underlying patient factors, such as osteoporosis, may exert greater influence on complication profiles than the number of treated vertebral segments [29–31]. Prior literature has documented that neurological sequelae, including transient motor weakness, thigh pain, and sensory disturbances, typically result from intraoperative neural tissue manipulation [23]. Furthermore, our observed tendency toward higher revision rates following multi-level procedures likely reflects the increased physiological stress and enhanced technical demands associated with more extensive spinal reconstructions.

Comparative analysis of fusion techniques for degenerative spondylolisthesis revealed no consistent clinical outcome differences [32]. For patients with additional stenotic segments, clinicians may select either isolated decompression or combined decompression with fusion. Our sub-analysis demonstrated no significant clinical differences between multi-level and single-level fusion approaches, though multi-level procedures were associated with increased intraoperative hemorrhage and extended operative duration.

Clinical practice varies considerably, when performing suprajacent decompression above a fused segment, the posterior tension band complex (spinous process-supraspinous ligament-spinous process) is frequently sacrificed, potentially compromising stability. The fused caudal segment may impose additional biomechanical stress on adjacent levels, potentially precipitating segmental instability [8, 33, 34]. Additionally, non-fused decompressed levels risk symptomatic bone regrowth [35, 36]. Conversely, multi-level fusion may accelerate adjacent segment pathology through increased biomechanical demand at remaining mobile segments [12, 13, 37, 38].

Despite these theoretical rationales, direct comparative evidence remains limited. In the SPORT cohort analysis by Smorgick et al., no statistically significant differences were observed between single- and multi-level fusion in clinical outcomes, recurrent stenosis, or reoperation rates at four-year follow-up [2]. They reported a higher frequency of pseudarthrosis-related exploration in the single-level group, which may have been influenced by greater use of non-instrumented techniques in that cohort (two of three pseudarthrosis cases occurred in the single-level arm) [2]. Importantly, their data consistently showed greater operative time and intraoperative blood loss with multi-level fusion, without corresponding differences in transfusion requirements, supporting the concept that fusion extension increases perioperative burden while offering limited measurable benefit in long-term outcomes [2].

In a separate SPORT trial subanalysis, Park and colleagues examined the impact of stenosis level extent on clinical outcomes. Their findings revealed that while stenosis level count did not predict outcomes in patients without spondylolisthesis, patients with degenerative spondylolisthesis and multi-level stenosis demonstrated inferior results across all primary and secondary outcome measures at two-year follow-up compared to those with single-level pathology [39].

Our findings regarding the advantages of single level over multi-level lumbar interbody fusion align with several previous studies. The significantly reduced operative time, blood loss, and hospital stay observed in our meta-analysis are consistent with established literature. Levin et al. similarly reported that single-level procedures required less operative time and had reduced blood loss compared to multi-level interventions [24]. Singh et al. also concluded that two-level ALIF was associated with higher procedural time and length of stay than one-level ALIF, supporting our findings [28].

The 41% reduction in revision surgery rates for single-level procedures is corroborated by Guo et al., who specifically noted that osteoporotic patients with double-level or multi-level pedicle screw fixation benefited less than those with single-level pedicle screw fixation [21]. Our finding of an 84% reduction in screw loosening complications with single-level procedures is directly supported by Guo’s observation that screw loosening rates were higher in the cranial and caudal vertebrae of multi-level constructs [21]. The superior functional outcomes (ODI scores) with single-level procedures in our analysis are partially supported by Harada et al., who reported that “patients in multi-level fusions experienced less improvement in back pain” compared to single-level posterolateral fusion patients [22].

Regarding ASD, our pooled analysis did not demonstrate a statistically significant difference between Single-Level and more extensive fusion constructs. This contrasts with Martini et al., who suggested that ASD risk after ALIF is driven primarily by the number of fused levels rather than demographic or intraoperative factors [11]. Differences in surgical approach, baseline alignment, patient selection, follow-up duration, and, critically, heterogeneity in ASD definitions (radiographic changes versus symptomatic ASD requiring reoperation) may explain these discrepant findings. Importantly, given the wide confidence intervals and generally low certainty of evidence, the absence of statistical significance in our analysis should not be interpreted as equivalence, but rather as insufficient evidence to confirm a difference in ASD risk between strategies.

Similarly, our analysis revealed no significant differences in infection rates between single and multi-level procedures, in contrast to Harada et al. [7], who reported that multi-level fusion patients “had more complications,” a category that typically includes infections. These divergent findings may be attributed to heterogeneity in study designs, including differences in patient demographics (e.g., age, comorbidities), surgical protocols (e.g., operative time, antibiotic prophylaxis), and follow-up durations.

The findings of this meta-analysis have significant implications for clinical practice in spine surgery. Surgeons should consider the substantial advantages of single-level lumbar interbody fusion over multi-level procedures when clinically appropriate. These benefits must be balanced against individual patient factors, particularly in cases where multi-level pathology exists. For patients with multi-level stenosis and single-level degenerative spondylolisthesis, the decision between isolated decompression versus combined decompression with fusion should be carefully evaluated, as our analysis suggests equivalent clinical outcomes despite the increased perioperative burden of multi-level procedures. Importantly, the absence of significant differences in adjacent segment deterioration between single and multi-level fusions challenges the rationale for extended fusion as prophylaxis against adjacent segment disease. These findings support a more selective approach to fusion levels, potentially limiting intervention to documented unstable segments while considering patient-specific factors such as bone quality, comorbidities, and functional demands.

Moreover, optimal fusion strategy must be guided not only by the extent of degenerative disease but also by a precise understanding of individual morpho-functional characteristics. This alignment between anatomical variability and surgical decision-making supports a more patient-specific approach, where fusion levels and fixation techniques are tailored to unique structural features. For instance, recent literature highlights the clinical relevance of accessory ossicles (e.g., Oppenheimer’s ossicle), which can mimic degenerative pathology. Advanced modalities such as CT or SPECT are frequently required to differentiate these ossicles from true pathological lesions. Incorporating this anatomical understanding is essential when interpreting postoperative symptoms, as precise recognition of such variants reduces the risk of misdiagnosis and prevents unnecessary fusion extension [40].

To our knowledge, this is the first meta-analysis comparing single-level interbody fusion versus double and multi-level interbody fusion procedures. By pooling data from 10 studies encompassing 1,430 patients, our findings provide robust evidence regarding the comparative outcomes of these procedures. This comprehensive analysis addresses an important gap in the literature and offers valuable insights for clinical decision-making in spinal surgery.

Despite these strengths, several limitations warrant consideration. First, including both single-level and multilevel degenerative disorders introduces clinical heterogeneity, as these conditions may differ in baseline symptom drivers, disease severity, alignment, comorbidity/frailty, bone quality, and natural history. Patients undergoing multilevel fusion may therefore represent a more advanced disease phenotype, raising the risk of confounding by indication; differences in revision risk and functional recovery may partly reflect baseline disease burden rather than fusion extent alone. Because baseline characteristics and indication-specific outcomes were inconsistently reported, we could not reliably stratify or adjust for these factors, and pooled estimates should be interpreted cautiously. Second, most included studies were retrospective, increasing susceptibility to selection bias, inconsistent data collection, and residual confounding. We could not stratify outcomes by surgical approach (e.g., PLIF, ALIF, LLIF), and heterogeneity in outcomes, follow-up duration, patient populations, techniques, implants, and perioperative protocols limits generalizability. Publication bias could not be reliably assessed because fewer than 10 studies were available for most outcomes. Therefore, pooled estimates primarily reflect the direction of effect and should be considered hypothesis-generating rather than confirmatory.

To reconcile these discrepancies, future prospective studies with standardized outcome measures, longer follow-up periods, and rigorous adjustment for confounding variables are warranted. Such studies would enhance the generalizability of findings and provide more definitive evidence regarding the comparative risks and benefits of single versus multi-level spinal fusion procedures.

## Conclusions

Single-level lumbar interbody fusion may offer perioperative advantages compared with more extensive fusion constructs, including fewer revisions, shorter operative time, reduced blood loss, shorter hospitalization, and fewer screw loosening events in pooled analyses. Functional outcomes generally favored single-level procedures, while several complications (including adjacent segment deterioration) did not differ significantly between groups. However, given low to very low certainty of evidence and substantial heterogeneity for several perioperative outcomes, these findings should be interpreted cautiously and individualized to patient pathology and surgical context. Future prospective studies with standardized outcome definitions, longer follow-up, and rigorous confounding control are needed to better define the optimal extent of fusion for multilevel degenerative disease.

## Appendix A

See Tables 2 and 3.

**Table 2:** Search Strategy with number retrieved from different data bases

Database	Search Query	Field	Results
PubMed	(“degenerative spondylolisthesis” OR “lumbar spondylolisthesis” OR “spinal stenosis” OR “degenerative disc disease” OR “lumbar degenerative disease” OR “degenerative lumbar spine”) AND (“single level” OR “Single level” OR “mono segmental” OR “mono level” OR “multi-level” OR “multi-level” OR “multiple-level” OR “bi-level” OR “three-level” OR “multi-segment” OR “multi-segmental”)	All	1652
Scopus	(“degenerative spondylolisthesis” OR “lumbar spondylolisthesis” OR “spinal stenosis” OR “degenerative disc disease” OR “lumbar degenerative disease” OR “degenerative lumbar spine”) AND (“single level” OR “Single level” OR “mono segmental” OR “mono level” OR “multi-level” OR “multi-level” OR “multiple-level” OR “bi-level” OR “three-level” OR “multi-segment” OR “multi-segmental”)	Title, abstract, keywords	2002
WOS	(“degenerative spondylolisthesis” OR “lumbar spondylolisthesis” OR “spinal stenosis” OR “degenerative disc disease” OR “lumbar degenerative disease” OR “degenerative lumbar spine”) AND (“single level” OR “Single level” OR “mono segmental” OR “mono level” OR “multi-level” OR “multi-level” OR “multiple-level” OR “bi-level” OR “three-level” OR “multi-segment” OR “multi-segmental”)	All	1579
Cochrane	(“degenerative spondylolisthesis” OR “lumbar spondylolisthesis” OR “spinal stenosis” OR “degenerative disc disease” OR “lumbar degenerative disease” OR “degenerative lumbar spine”) AND (“single level” OR “Single level” OR “mono segmental” OR “mono level” OR “multi-level” OR “multi-level” OR “multiple-level” OR “bi-level” OR “three-level” OR “multi-segment” OR “multi-segmental”)	All	418
Total			5651

**Table 3:** ROBINS-I risk of bias assessment for included non-randomized studies

Study (design)	Bias due to confounding	Selection of participants	Classification of interventions	Deviations from intended interventions	Missing data	Measurement of outcomes	Selection of reported result	Overall ROBINS-I judgment
Guo 2020 (retrospective cohort)	**Moderate** – retrospective, non-randomized; limited adjustment (age/BMI), but groups appear broadly comparable for major clinical indications.	**Moderate** – hospital-based cohort, likely consecutive but not clearly population-based.	**Low** – interbody fusion level clearly defined from operative records.	**Low** – no evidence that deviations from the planned surgery differed between groups.	**Moderate** – ~32-month follow-up; losses to follow-up not fully characterized but likely acceptable.	**Low** – revision, screw loosening, and radiographic outcomes objectively defined and measured.	**Moderate** – no prespecified protocol; selective reporting cannot be excluded but main outcomes are presented.	**Moderate**
Hiyama 2025 (retrospective cohort)	**Moderate** – no formal multivariable model, but baseline characteristics are described and groups are reasonably similar for key clinical factors.	**Moderate** – single-centre LDD cohort with clear inclusion criteria; generalizability somewhat limited.	**Low** – single- vs. multi-level LLIF clearly classified.	**Low** – operations followed routine clinical care; no indication of differential co-interventions.	**Moderate** – 12-month follow-up; attrition and handling of missing data not fully detailed but appear modest.	**Low** – pain/QoL and complications measured with standard, validated instruments.	**Moderate** – relevant outcomes reported, though without prospective registration or analysis plan.	**Moderate**
Levin 2007 (retrospective cohort)	**Moderate–Serious** – small sample with no formal adjustment, but indication and technique relatively homogeneous.	**Moderate** – elective DDD cases meeting explicit criteria; possible selection of younger/healthier patients.	**Low** – 1- vs. 2-level procedures clearly distinguished.	**Low** – no evidence of systematic deviations from planned surgery.	**Moderate** – ~30-month follow-up; completeness and handling of missing data not fully described but follow-up appears reasonable.	**Low** – operative time, blood loss, and charges abstracted from objective records.	**Moderate** – financial and clinical outcomes likely chosen post hoc, but key perioperative endpoints are presented.	**Moderate**
Martini 2020 (retrospective cohort)	**Moderate** – no detailed adjustment for sagittal alignment, osteoporosis, or comorbidity; baseline descriptions suggest broadly comparable groups.	**Moderate** – 1–2 level ALIF for DDD over a defined timeframe at a single centre; reasonably representative of that practice.	**Low** – number of fused levels and ALIF procedures clearly documented.	**Low** – no evidence of systematic deviations from the intended surgical approach.	**Moderate** – mid-term follow-up; completeness acceptable but not fully quantified.	**Low** – ASD and reoperations identified using standard clinical and radiographic criteria.	**Moderate** – focus on ASD; other endpoints less completely reported, but main outcomes for the review question are available.	**Moderate**
Sakaura 2013 (retrospective cohort)	**Serious** – no control for major confounders (age, bone quality, baseline disability, comorbidities) in DS patients.	**Serious** – small single-centre series under one surgeon’s strategy with limited representativeness and clear potential for selection bias.	**Low** – 1- vs. 2-level PLIF clearly distinguished in operative records.	**Moderate** – some evolution in technique over time is possible and not fully described.	**Serious** – completeness of follow-up and handling of missing data poorly documented.	**Low–Moderate** – clinical outcomes and ASD largely objective but not blinded.	**Serious** – high risk of selective outcome reporting in a small retrospective series.	**Serious**
Singh 2025 (retrospective cohort)	**Moderate** – some adjustment performed; residual confounding by deformity, alignment, or bone quality cannot be excluded but is partly addressed.	**Moderate** – adult degenerative spine patients undergoing elective ALIF at a single centre; typical of tertiary practice but not population-based.	**Low** – 1- vs. 2-level ALIF clearly defined.	**Low** – no indication of systematic deviations or differential co-interventions.	**Moderate** – follow-up focused on short- to mid-term PROMs; attrition not fully detailed but appears acceptable.	**Low** – PROMs and perioperative variables collected with standard methods.	**Moderate** – emphasis on PROMs and perioperative metrics, without pre-registration.	**Moderate**
Smorgick 2014 (SPORT subanalysis)	**Moderate** – subset comparison within SPORT with some adjustment; residual confounding likely but partially mitigated by the parent study design.	**Moderate** – patients from SPORT centres with explicit inclusion criteria; selection issues mainly arise from using a subset for this analysis.	**Low** – surgical strategy (single vs. multilevel fusion) classified from operative records.	**Low** – deviations from intended surgery unlikely to differ systematically between groups.	**Moderate** – 4-year follow-up with reasonable retention, though some attrition remains.	**Low** – standardized SPORT outcome assessments and imaging protocols.	**Moderate** – SPORT had a prespecified framework, but this subanalysis was not pre-registered.	**Moderate**
Barrett-Tuck 2017 (retrospective cohort)	**Serious** – small retrospective series with no confounder control and heterogeneity in devices and indications.	**Serious** – highly selected patients with persistent symptoms treated with device-based PLIF; strong selection bias likely.	**Low** – single- vs. two-level PLIF clearly defined.	**Moderate** – learning curve and co-interventions not clearly documented.	**Serious** – 12-month follow-up; completeness and handling of missing data unclear in a small cohort.	**Low–Moderate** – symptom relief and fusion assessed clinically and radiographically, without blinding.	**Serious** – selective outcome reporting likely in a small case series.	**Serious**
Harada 2021 (retrospective cohort)	**Moderate** – no comprehensive multivariable model, but baseline characteristics are reasonably described and similar between groups.	**Moderate** – consecutive low-grade DS patients at a single centre; fairly typical of clinical practice but not population-based.	**Low** – number of fused levels clearly documented.	**Low** – no evidence that deviations or co-interventions differed between groups.	**Moderate** – 6-month follow-up reported; longer-term outcomes partially described, with acceptable core follow-up.	**Low** – pain, complications, and discharge destination obtained from charts and PROs using standard methods.	**Moderate** – main outcomes relevant to the research question are reported despite lack of protocol.	**Moderate**

## Appendix B

**Fig. 9:**
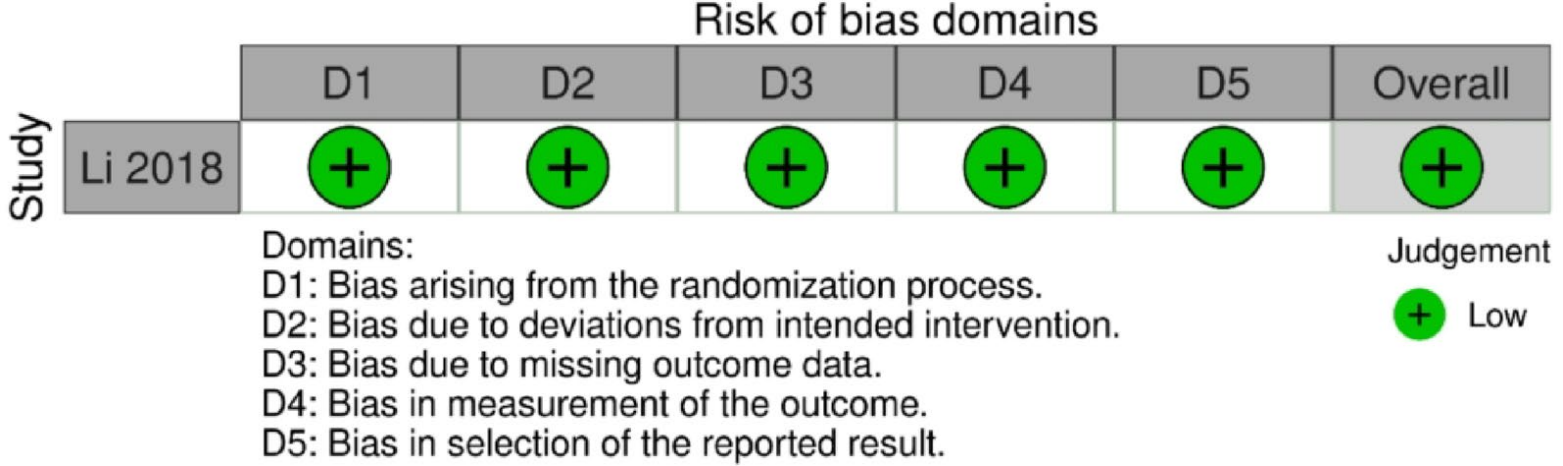
Cochrane risk of bias assessment tool 2 for randomized control trail

**Fig. 10:**
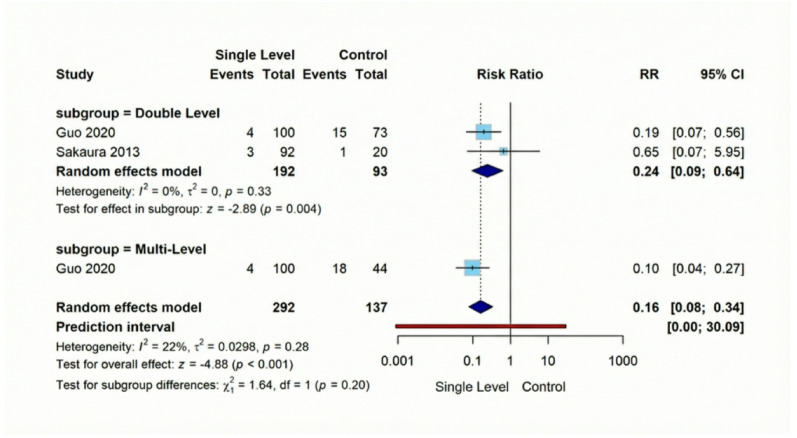
Forest plot for random effect model meta-analysis for screw loosening rates outcome

**Fig. 11:**
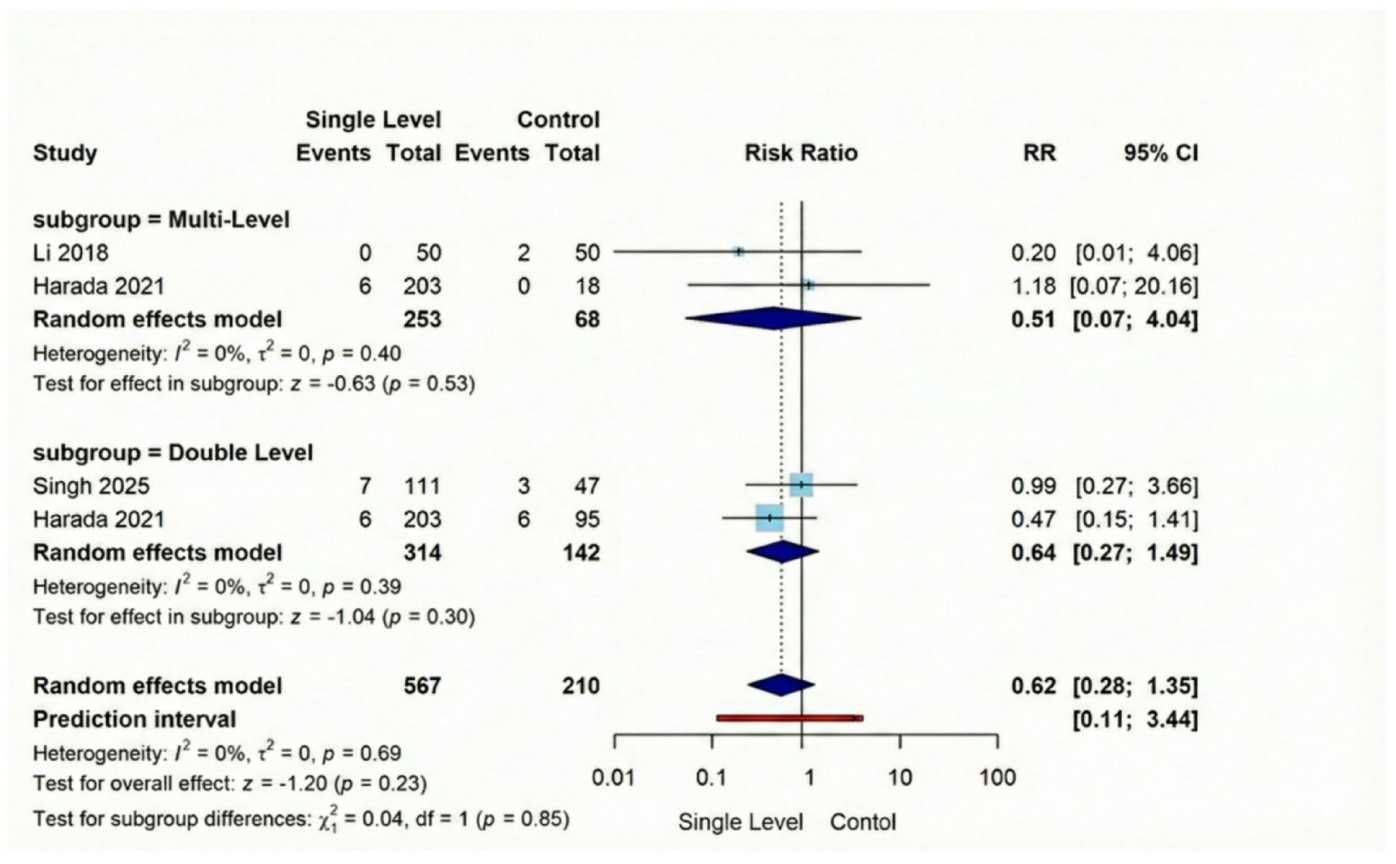
Forest plot for random effect model meta-analysis for adjacent segment disease outcome

**Fig. 12:**
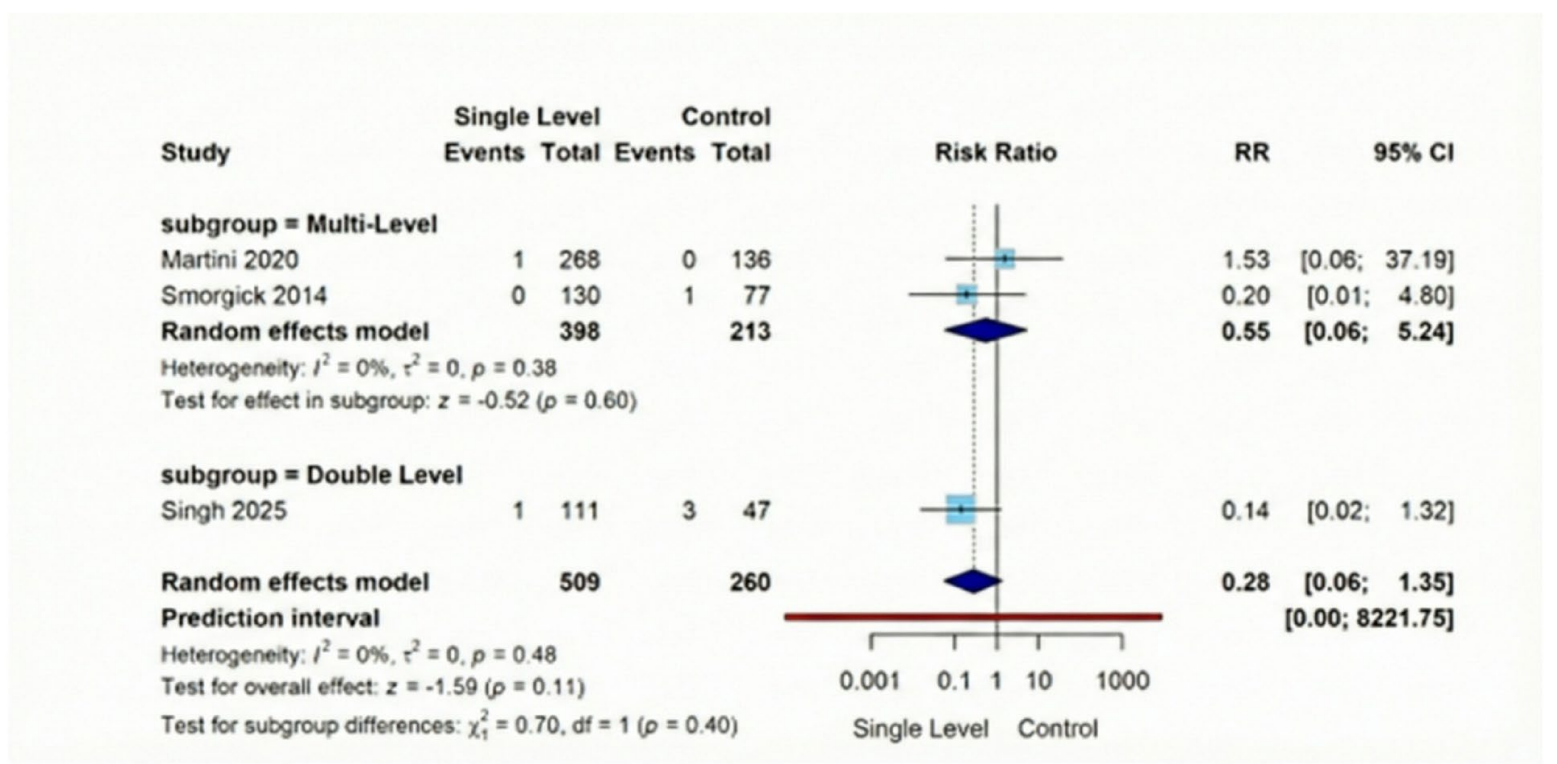
Forest plot for random effect model meta-analysis for vascular injury outcome

**Fig. 13:**
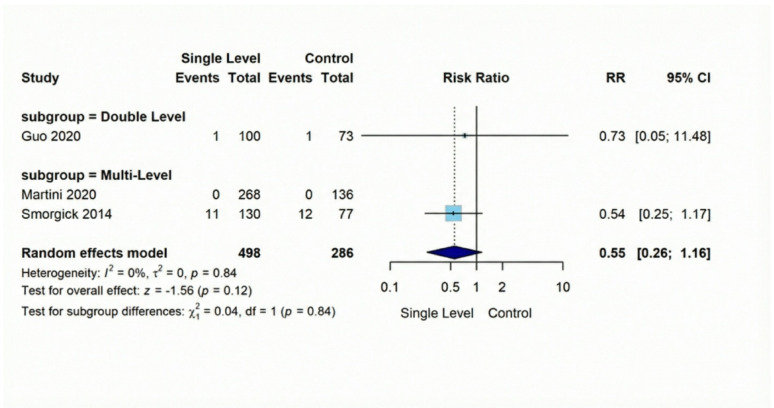
Forest plot for random effect model meta-analysis for dural tear outcome

See Figs. 9, 10, 11, 12 and 13.

## Supplementary Information

Below is the link to the electronic supplementary material.

Supplementary Material 1

Supplementary Material 2

## Data Availability

The original contributions presented in this study are included in the article/supplementary material. Further inquiries can be directed to the corresponding author.

## Abbreviations

DS: Degenerative spondylolisthesis
SPORT: Spine patient outcomes research trial
PLIF: Posterior lumbar interbody fusion
ALIF: Anterior lumbar interbody fusion
LLIF: Lateral lumbar interbody fusion
ODI: Oswestry disability index

## References

[1] Ravindra VM, Senglaub SS, Rattani A, Dewan MC, Härtl R, Bisson E, et al. Degenerative Lumbar spine disease: estimating global incidence and worldwide volume. Glob Spine J. 2018;8:784–94. 10.1177/2192568218770769. 30560029 10.1177/2192568218770769 PMC6293435

[2] Smorgick Y, Park DK, Baker KC, Lurie JD, Tosteson TD, Zhao W, et al. Single- versus multi-level fusion for single-level degenerative spondylolisthesis and multi-level lumbar stenosis: four-year results of the spine patient outcomes research trial. Spine. 2013;38:797–805. 10.1097/BRS.0b013e31827db30f. 23169068 10.1097/BRS.0b013e31827db30f PMC3757550

[3] Minamide A, Yoshida M, Simpson AK, Nakagawa Y, Iwasaki H, Tsutsui S, et al. Minimally invasive spinal decompression for degenerative lumbar spondylolisthesis and stenosis maintains stability and may avoid the need for fusion. Bone Joint J. 2018;100–B:499–506. 10.1302/0301-620X.100B4.BJJ-2017-0917.R1. 29629597 10.1302/0301-620X.100B4.BJJ-2017-0917.R1

[4] Eismont FJ, Norton RP, Hirsch BP. Surgical management of lumbar degenerative spondylolisthesis. J Am Acad Orthop Surg. 2014;22:203–13. 10.5435/JAAOS-22-04-203. 24668350 10.5435/JAAOS-22-04-203

[5] Herkowitz HN, Kurz LT. Degenerative lumbar spondylolisthesis with spinal stenosis. A prospective study comparing decompression with decompression and intertransverse process arthrodesis. J Bone Joint Surg Am. 1991;73:802–8. 2071615

[6] Ghogawala Z, Dziura J, Butler WE, Dai F, Terrin N, Magge SN, et al. Laminectomy plus fusion versus laminectomy alone for lumbar spondylolisthesis. N Engl J Med. 2016;374:1424–34. 10.1056/NEJMoa1508788. 27074067 10.1056/NEJMoa1508788

[7] Weinstein JN, Lurie JD, Tosteson TD, Hanscom B, Tosteson ANA, Blood EA, et al. Surgical versus nonsurgical treatment for lumbar degenerative spondylolisthesis. N Engl J Med. 2007;356:2257–70. 10.1056/NEJMoa070302. 17538085 10.1056/NEJMoa070302 PMC2553804

[8] Subramaniam V, Chamberlain RH, Theodore N, Baek S, Safavi-Abbasi S, Senoğlu M, et al. Biomechanical effects of laminoplasty versus laminectomy: stenosis and stability. Spine (Phila Pa 1976). 2009;34:E573–578. 10.1097/BRS.0b013e3181aa0214. 19770600 10.1097/BRS.0b013e3181aa0214

[9] Nagata H, Schendel MJ, Transfeldt EE, Lewis JL. The effects of immobilization of long segments of the spine on the adjacent and distal facet force and lumbosacral motion. Spine.1993;8:2471–9. 10.1097/00007632-199312000-00017. 10.1097/00007632-199312000-00017 8303451

[10] Ryu R, Techy F, Varadarajan R, Amirouche F. Effect of interbody fusion on the remaining discs of the lumbar spine in subjects with disc degeneration. Orthop Surg. 2016;8:27–33. 10.1111/os.12219. 27028378 10.1111/os.12219 PMC6584392

[11] Lee S, Pandher D, Yoon K, Lee S, Oh KJ. The effect of postoperative immobilization on short-segment fixation without bone grafting for unstable fractures of thoracolumbar spine. Indian J Orthop. 2009;43:197–204. 10.4103/0019-5413.41870. 19838371 10.4103/0019-5413.41870 PMC2762247

[12] Chow DH, Luk KD, Evans JH, Leong JC. Effects of short anterior lumbar interbody fusion on biomechanics of neighboring unfused segments. Spine (Phila Pa 1976). 1996;21:549–55. 10.1097/00007632-199603010-00004. 8852308 10.1097/00007632-199603010-00004

[13] Chen CS, Cheng CK, Liu CL, Lo WH. Stress analysis of the disc adjacent to interbody fusion in lumbar spine. Med Eng Phys. 2001;23:483–91. 10.1016/s1350-4533(01)00076-5. 11574255 10.1016/s1350-4533(01)00076-5

[14] Johnsson KE, Willner S, Johnsson K. Postoperative instability after decompression for lumbar spinal stenosis. Spine (Phila Pa 1976). 1986;11:107–10. 10.1097/00007632-198603000-00001. 3704799 10.1097/00007632-198603000-00001

[15] Liberati A. The PRISMA statement for reporting systematic reviews and meta-analyses of studies that evaluate healthcare interventions: explanation and elaboration. BMJ. 2009;339:2700–2700. 10.1136/bmj.b2700. 10.1136/bmj.b2700 PMC2714672 19622552

[16] Higgins JPT, Thomas J, Chandler J, Cumpston M, Li T, Page MJ, Welch VA, editors. Cochrane Handbook for Systematic Reviews of Interventions. 2nd ed. Chichester (UK): Wiley; 2019.

[17] Sterne JA, Hernán MA, Reeves BC, Savović J, Berkman ND, Viswanathan M, et al. ROBINS-I: a tool for assessing risk of bias in non-randomised studies of interventions. BMJ. 2016;355:i4919. 10.1136/bmj.i4919. 27733354 10.1136/bmj.i4919 PMC5062054

[18] Sterne JAC, Savović J, Page MJ, Elbers RG, Blencowe NS, Boutron I, et al. RoB 2: a revised tool for assessing risk of bias in randomised trials. BMJ. 2019;366:l4898. 10.1136/bmj.l4898. 31462531 10.1136/bmj.l4898

[19] Guyatt GH, Oxman AD, Vist GE, Kunz R, Falck-Ytter Y, Alonso-Coello P, et al. GRADE: an emerging consensus on rating quality of evidence and strength of recommendations. BMJ. 2008;336:924–6. 10.1136/bmj.39489.470347.AD. 18436948 10.1136/bmj.39489.470347.AD PMC2335261

[20] Barrett-Tuck R, Del Monaco D, Block JE. One and two level posterior lumbar interbody fusion (PLIF) using an expandable, stand-alone, interbody fusion device: a VariLift^®^ case series. J Spine Surg. 2017;3:9–15. 10.21037/jss.2017.02.05. 28435912 10.21037/jss.2017.02.05 PMC5386905

[21] Guo H, Tang Y, Guo D, Ma Y, Yuan K, Li Y, et al. Pedicle screw fixation in single-level, double-level, or multi-level posterior lumbar fusion for osteoporotic spine: a retrospective study with a minimum 2-year follow-up. World Neurosurg. 2020;140:e121–8. 10.1016/j.wneu.2020.04.198. 32376379 10.1016/j.wneu.2020.04.198

[22] Harada GK, Khan JM, Vetter C, Basques BA, Sayari AJ, Hayani Z, et al. Does the number of levels fused affect spinopelvic parameters and clinical outcomes following posterolateral lumbar fusion for low-grade spondylolisthesis? Glob Spine J. 2021;11:116–21. 10.1177/2192568220901527. 32875855 10.1177/2192568220901527 PMC7734270

[23] Hiyama A, Sakai D, Katoh H, Sato M, Watanabe M. Evaluating single-level vs. multi-level lateral lumbar interbody fusion: clinical outcomes and complications. J Clin Neurosci. 2025;134:111082. 10.1016/j.jocn.2025.111082. 39893929 10.1016/j.jocn.2025.111082

[24] Levin DA, Bendo JA, Quirno M, Errico T, Goldstein J, Spivak J. Comparative charge analysis of one-and two-level lumbar total disc arthroplasty versus circumferential lumbar fusion. Spine. 2007;32:2905–9. 10.1097/BRS.0b013e31815b84ae 10.1097/BRS.0b013e31815b84ae 18246016

[25] Li T, Shi L, Luo Y, Chen D, Chen Y. One-level or multi-level interbody fusion for multi-level lumbar degenerative diseases: a prospective randomized control study with a 4-year follow-Up. World Neurosurg. 2018;110:e815–22. 10.1016/j.wneu.2017.11.109. 29191543 10.1016/j.wneu.2017.11.109

[26] Martini ML, Nistal DA, Gal J, Neifert SN, Rothrock RJ, Kim JD, et al. Adjacent segment reoperation and other perioperative outcomes in patients who underwent anterior lumbar interbody fusions at one and two levels. World Neurosurg. 2020;139:e480–8. 10.1016/j.wneu.2020.04.053. 32311547 10.1016/j.wneu.2020.04.053

[27] Sakaura H, Yamashita T, Miwa T, Ohzono K, Ohwada T. Outcomes of 2-level posterior lumbar interbody fusion for 2-level degenerative lumbar spondylolisthesis: Clinical article. SPI. 2013;19:90–4. 10.3171/2013.4.SPINE12651. 10.3171/2013.4.SPINE12651 23662887

[28] Singh M, Knebel A, Kuharski MJ, Nassar JE, Callanan T, Basques BA, et al. One-Level versus two-level anterior lumbar interbody fusion (ALIF) from L4 to S1: comparison of complications, alignment, and patient outcomes. Spine. 2025;50:271–6. 10.1097/BRS.0000000000005133. 39192751 10.1097/BRS.0000000000005133

[29] Wu H, Shan Z, Zhao F, Cheung JPY. Poor bone quality, multi-level surgery, and narrow and tall cages are associated with intraoperative endplate injuries and late-onset cage subsidence in lateral lumbar interbody fusion: a systematic review. Clin Orthop Relat Res. 2022;480:163–88. 10.1097/CORR.0000000000001915. 34324459 10.1097/CORR.0000000000001915 PMC8673985

[30] Hiyama A, Sakai D, Katoh H, Sato M, Watanabe M. Impact of osteoporosis on short-term surgical outcomes in lumbar degenerative disease patients undergoing lateral lumbar interbody fusion: a retrospective analysis. World Neurosurg. 2024;188:e424–33. 10.1016/j.wneu.2024.05.130. 38802060 10.1016/j.wneu.2024.05.130

[31] Tempel ZJ, Gandhoke GS, Okonkwo DO, Kanter AS. Impaired bone mineral density as a predictor of graft subsidence following minimally invasive transpsoas lateral lumbar interbody fusion. Eur Spine J. 2015;24:414–9. 10.1007/s00586-015-3844-y. 25739988 10.1007/s00586-015-3844-y

[32] Abdu WA, Lurie JD, Spratt KF, Tosteson ANA, Zhao W, Tosteson TD, et al. Degenerative spondylolisthesis: does fusion method influence outcome? Four-year results of the spine patient outcomes research trial. Spine (Phila Pa 1976). 2009;34:2351–60. 10.1097/BRS.0b013e3181b8a829. 19755935 10.1097/BRS.0b013e3181b8a829 PMC3750746

[33] Lu WW, Luk KD, Ruan DK, Fei ZQ, Leong JC. Stability of the whole lumbar spine after multi-level fenestration and discectomy. Spine (Phila Pa 1976). 1999;24:1277–82. 10.1097/00007632-199907010-00002. 10404567 10.1097/00007632-199907010-00002

[34] Tai C-L, Hsieh P-H, Chen W-P, Chen L-H, Chen W-J, Lai P-L. Biomechanical comparison of lumbar spine instability between laminectomy and bilateral laminotomy for spinal stenosis syndrome: an experimental study in porcine model. BMC Musculoskelet Disord. 2008;9:84. 10.1186/1471-2474-9-84. 18547409 10.1186/1471-2474-9-84 PMC2438358

[35] Postacchini F, Cinotti G. Bone regrowth after surgical decompression for lumbar spinal stenosis. J Bone Joint Surg Br. 1992;74:862–9. 10.1302/0301-620X.74B6.1447247. 1447247 10.1302/0301-620X.74B6.1447247

[36] Turcotte JJ, Patton CM. Predictors of postoperative complications after surgery for lumbar spinal stenosis and degenerative lumbar spondylolisthesis. J Am Acad Orthop Surg Glob Res Rev. 2018;2:e085. 10.5435/JAAOSGlobal-D-18-00085. 30680370 10.5435/JAAOSGlobal-D-18-00085 PMC6336577

[37] Auerbach JD, Lonner BS, Errico TJ, Freeman A, Goerke D, Beaubien BP. Quantification of intradiscal pressures below thoracolumbar spinal fusion constructs: is there evidence to support saving a level? Spine (Phila Pa 1976). 2012;37:359–66. 10.1097/BRS.0b013e31821e1106. 21540780 10.1097/BRS.0b013e31821e1106

[38] Ignasiak D, Peteler T, Fekete TF, Haschtmann D, Ferguson SJ. The influence of spinal fusion length on proximal junction biomechanics: a parametric computational study. Eur Spine J. 2018;27:2262–71. 10.1007/s00586-018-5700-3. 30039253 10.1007/s00586-018-5700-3

[39] Park DK, An HS, Lurie JD, Zhao W, Tosteson A, Tosteson TD, et al. Does multi-level lumbar stenosis lead to poorer outcomes? A subanalysis of the Spine patient outcomes research trial (SPORT) lumbar stenosis study. Spine (Phila Pa 1976). 2010;35:439–46. 10.1097/BRS.0b013e3181bdafb9. 20081560 10.1097/BRS.0b013e3181bdafb9 PMC2886146

[40] Ogut E, Karakas O, Aydin DD. Oppenheimer’s accessory ossicle and clinical significance: a narrative review. J Orthop Rep. 2022;1(4):100069. 10.1016/j.jorep.2022.100069.

