# Supplementary material for: Single level versus multi-level lumbar interbody fusion for lumbar degenerative diseases: a systematic review and meta analysis
Source: J Orthop Surg Res. 2026 Mar 14;21:273. doi: 10.1186/s13018-026-06778-4 (PMC13104495; doi:10.1186/s13018-026-06778-4)
Supplement: Supplementary file 1 — Supplementary Material 1 [file 13018_2026_6778_MOESM1_ESM.docx]

**Supplementary Appendix C: List of Excluded Full-Text Articles**

The following **27 studies** were assessed full-text but were excluded from the final analysis. Studies excluded as conference abstracts (n=47), reviews (n=33), foreign language (n=41), or animal studies (n=17) are not listed below. This list comprises studies excluded for specific methodological reasons (e.g., wrong comparison, wrong population, or duplicate data).

| # | Study ID (Author, Year) | Reason for Exclusion | Study Link |
| --- | --- | --- | --- |
| 1 | Gerling et al. (2010) | Duplicate Cohort: Sub-analysis of the SPORT trial; data overlaps with included study (Smorgick et al., 2013). | [PubMed Link](https://pubmed.ncbi.nlm.nih.gov/26656062/) |
| 2 | Abdu et al. (2009) | Wrong Comparison: Compared PLF vs. PLIF/360 fusion (Technique comparison, not Single vs. Multi-level). | [PubMed Link](https://pubmed.ncbi.nlm.nih.gov/19755935/) |
| 3 | Weinstein et al. (2007) | Wrong Comparison: Compared Surgical vs. Non-surgical treatment (SPORT trial primary analysis). | [PubMed Link](https://pubmed.ncbi.nlm.nih.gov/17538085/) |
| 4 | Ghogawala et al. (2016) | Wrong Comparison: Compared Decompression vs. Fusion (SLIP Study), not Single vs. Multi-level fusion. | [PubMed Link](https://pubmed.ncbi.nlm.nih.gov/27074067/) |
| 5 | Försth et al. (2016) | Wrong Comparison: Compared Decompression vs. Fusion; did not stratify fusion levels as primary comparator. | [PubMed Link](https://pubmed.ncbi.nlm.nih.gov/27074066/) |
| 6 | Kilinçer et al. (2005) | Wrong Comparison: Compared outcomes in elderly vs. young patients; did not stratify by number of fusion levels. | [PubMed Link](https://pubmed.ncbi.nlm.nih.gov/16122020) |
| 7 | Nerland et al. (2016) | Wrong Intervention: Compared Laminectomy (Decompression) vs. Minimally Invasive Decompression. | [PubMed Link](https://pubmed.ncbi.nlm.nih.gov/25833966/) |
| 8 | Houra et al. (2022) | Wrong Population: Study focused on single-level foraminal stenosis without a multi-level control group. | [PubMed Link](https://pubmed.ncbi.nlm.nih.gov/35177520) |

| # | Study ID (Author, Year) | Reason for exclusion | Link |
| --- | --- | --- | --- |
| 9 | **Sulaiman et al., 2014** | Wrong comparison: compares MIS vs open TLIF; fusion levels mixed and not stratified as single vs multi-level. | <https://pubmed.ncbi.nlm.nih.gov/24688330/> (full text: <https://pmc.ncbi.nlm.nih.gov/articles/PMC3963049/>) |
| 10 | **Hartmann et al., 2022** | Wrong comparison: evaluates MIS vs open TLIF; does not compare single vs multi-level fusion as primary exposure. | <https://pmc.ncbi.nlm.nih.gov/articles/PMC9492567/> |
| 11 | **Wang et al., 2011** | Wrong population: revision TLIF after prior discectomy/laminectomy; not primary degenerative cases; no single vs multi-level comparison. | <https://pmc.ncbi.nlm.nih.gov/articles/PMC3065602/> |
| 12 | **Rampersaud et al., 2011** | Wrong outcome/comparison: economic cost-utility of MIS vs open fusion; no perioperative or clinical outcomes by number of levels fused. | <https://pmc.ncbi.nlm.nih.gov/articles/PMC4365621/> |
| 13 | **Goldstein et al., 2016** | Study type & wrong comparison: systematic review of MIS vs open posterior/TLIF approaches; not original data comparing single vs multi-level fusion. | <https://pubmed.ncbi.nlm.nih.gov/26825793/> |
| 14 | **Tosteson et al., 2008** | Wrong outcome: cost-effectiveness analysis of SPORT data; clinical results overlap primary SPORT papers; no dedicated single vs multi-level fusion analysis. | <https://pubmed.ncbi.nlm.nih.gov/19075203/> |
| 15 | **Goz et al., 2014** | Database study / wrong outcome: NIS administrative database; focuses on approach (ALIF, P/TLIF, AP) and costs; no granular ODI/clinical outcomes or single vs multi-level comparison. | <https://pubmed.ncbi.nlm.nih.gov/24333459/> |
| 16 | **Chen et al., 2022** | Wrong outcome: primary endpoint is 30-day readmission; does not provide detailed operative/functional outcomes by fusion level. | <https://pmc.ncbi.nlm.nih.gov/articles/PMC9972270/> |
| 17 | **Garcia et al., 2017** | Wrong comparison/outcome: only single-level TLIF; focuses on readmission risk factors; no multi-level control group and limited perioperative detail for your PICO. | <https://pmc.ncbi.nlm.nih.gov/articles/PMC5476349/> |
| 18 | **Reyes et al., 2020** | Database study: national trends and safety metrics; no level-specific clinical or functional outcomes and no explicit single vs multi-level comparison. | <https://pmc.ncbi.nlm.nih.gov/articles/PMC8113062/> |
| 19 | **Radcliff et al., 2013** | Wrong outcome/focus: narrative review of ASD after various procedures; does not provide direct single vs multi-level fusion outcome comparison. | <https://pubmed.ncbi.nlm.nih.gov/23773433/> |
| 20 | **Saavedra-Pozo et al., 2014** | Study type/outcome: review article on ASD pathophysiology and risk; no primary comparative cohort data for single vs multi-level lumbar fusion. | <https://pmc.ncbi.nlm.nih.gov/articles/PMC3963057/> |
| 21 | **Trivedi et al., 2018** | Wrong outcome: evidence-based review of ASD/ASDeg; focuses on degeneration rather than perioperative/functional outcomes by fusion level. | <https://pmc.ncbi.nlm.nih.gov/articles/PMC5810899/> |
| 22 | **Ye et al., 2021** | Wrong outcome: evaluates incidence/risk factors for ASD after TLIF; no direct comparison of single vs multi-level fusion in terms of perioperative/functional outcomes. | <https://pmc.ncbi.nlm.nih.gov/articles/PMC8604649/> |
| 23 | **Rothenfluh et al., 2015** | Radiographic/outcome mismatch: focuses on spinopelvic parameters and ASD risk; no perioperative metrics or explicit single vs multi-level comparison. | <https://pubmed.ncbi.nlm.nih.gov/25018033/> |
| 24 | **Pinto et al., 2021** | Wrong outcome: investigates surgical risk factors for ASD after lumbar fusion; does not directly compare single vs multi-level fusions on clinical endpoints. | <https://eor.bioscientifica.com/view/journals/eor/6/10/2058-5241.6.210050.xml> |
| 25 | **Matsumoto et al., 2017** | Radiographic risk-factor study: analyzes sagittal alignment and ASD; no primary comparison of outcomes between single vs multi-level fusion constructs. | <https://thejns.org/spine/view/journals/j-neurosurg-spine/26/4/article-p435.xml> |
| 26 | **Kim et al., 2015** | Wrong outcome/radiographic focus: evaluates lumbar lordosis–pelvic incidence gap and ASD; no detailed perioperative/functional comparison by number of levels fused. | <https://krspine.org/DOIx.php?id=10.4184%2Fjkss.2015.22.3.69> |
| 27 | **Djurasovic et al., 2019** | Wrong outcome: economic/cost-effectiveness analysis of a specific MIS technique; no explicit single vs multi-level comparison with detailed clinical outcomes. | <https://thejns.org/spine/view/journals/j-neurosurg-spine/32/1/article-p31.xml> |
