# Supplementary material for: Single level versus multi-level lumbar interbody fusion for lumbar degenerative diseases: a systematic review and meta analysis
Source: J Orthop Surg Res. 2026 Mar 14;21:273. doi: 10.1186/s13018-026-06778-4 (PMC13104495; doi:10.1186/s13018-026-06778-4)
Supplement: Supplementary file 2 — Supplementary Material 2 [file 13018_2026_6778_MOESM2_ESM.docx]

**Supplementary Table 1:** GRADE certainty of evidence for key outcomes

| Outcome | Effect (single- vs multi-level) | Certainty of evidence (GRADE) | Rationale for rating |
| --- | --- | --- | --- |
| Revision surgery | RR 0.59 (95% CI 0.40–0.86) – single-level fusion associated with ~41% lower revision risk. | Low | Body of evidence dominated by observational cohorts at serious risk of confounding (ROBINS-I serious for all NRS). Heterogeneity low (I²=7%) and CI excludes no effect, so no additional downgrade for inconsistency or imprecision, but overall certainty remains low because of non-randomized design and residual confounding. |
| Oswestry Disability Index (ODI) | MD –3.90 points (95% CI –7.89 to 0.10) – trend favouring single-level but CI includes no effect. | Very low | Started at low (observational evidence at serious risk of bias); downgraded for inconsistency (substantial heterogeneity, I²=66%) and imprecision (CI crosses the null and includes both small benefit and no important difference). Clinical importance of the pooled effect is uncertain. |
| Operative time | MD –60.73 minutes (95% CI –80.89 to –40.57) – shorter procedures with single-level fusion. | Very low | Direction of effect consistent (single-level always faster), but extreme heterogeneity (I²=96%) suggests large between-study differences in case-mix and surgical technique. Observational design and residual confounding plus substantial inconsistency reduce certainty to very low. |
| Intraoperative blood loss | MD –286.99 mL (95% CI –496.71 to –77.27) – lower blood loss with single-level fusion. | Very low | Effect strongly favours single-level, but I²=99% with very wide between-study variability. Observational design, serious risk of bias, and extreme heterogeneity lead to very low certainty. |
| Length of hospital stay | MD –1.22 days (95% CI –2.09 to –0.34) – shorter LOS after single-level fusion. | Very low | Effect direction consistent but substantial heterogeneity (I²=78%), differences in discharge practices, and non-randomized design. Downgraded for inconsistency and indirectness (health-system differences), yielding very low certainty. |
| Incision infection | RR 0.49 (95% CI 0.19–1.25) – no clear difference. | Very low | Events were infrequent; CIs are wide and include both important reduction and little/no difference. Observational design with serious risk of bias and imprecision from low event counts lead to very low certainty. |
| Screw loosening | RR 0.16 (95% CI 0.08–0.34) – ~84% lower risk with single-level constructs. | Low | Consistent direction across three studies with low heterogeneity (I²=22%). However, all data come from observational cohorts in which screw loosening is confounded by bone quality and construct length (ROBINS-I serious risk of bias), so certainty remains low despite the large effect. |
| Adjacent segment deterioration (ASD) | RR 0.62 (95% CI 0.28–1.35) – no statistically significant difference. | Very low | Observational data at serious risk of bias; CIs allow for both meaningful reduction and possible increase in ASD risk; follow-up and definitions of ASD vary across studies. Downgraded for imprecision and indirectness (mixed indications and constructs), leading to very low certainty. |


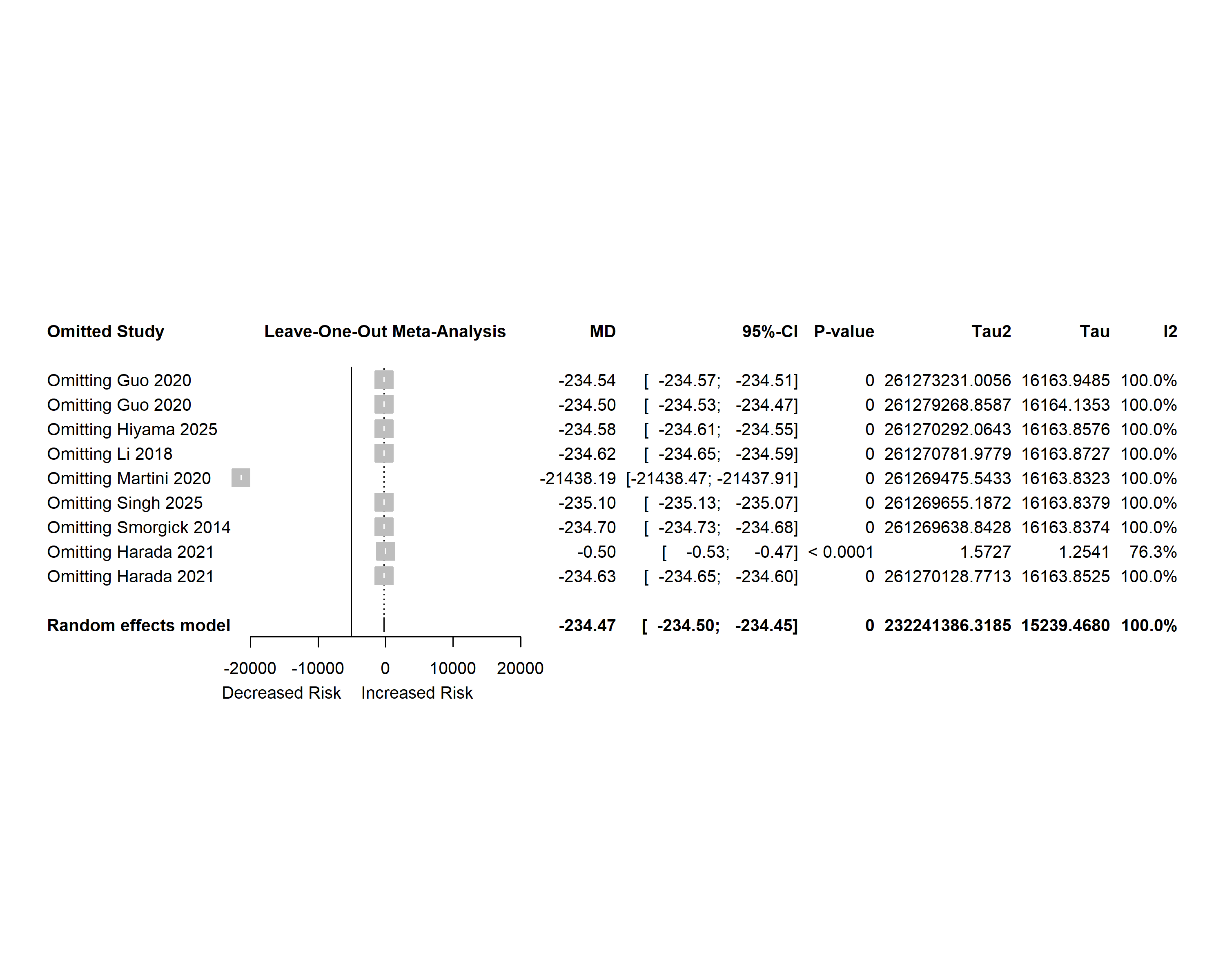

**Supplementary Figure 1:** Leave-one-out random-effects meta-analysis for postoperative length of stay

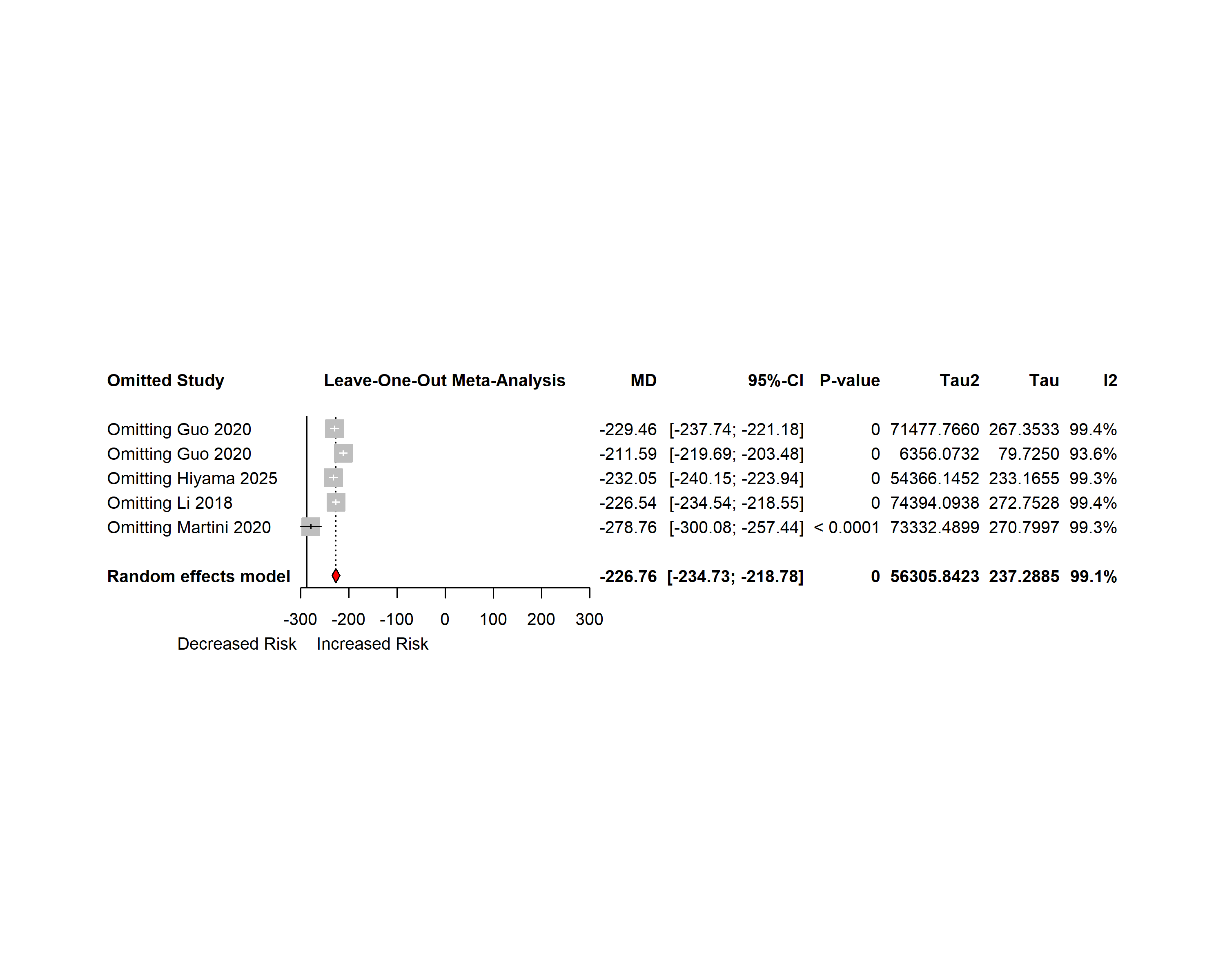

**Supplementary Figure 2:** Leave-one-out random-effects meta-analysis for intraoperative blood loss.


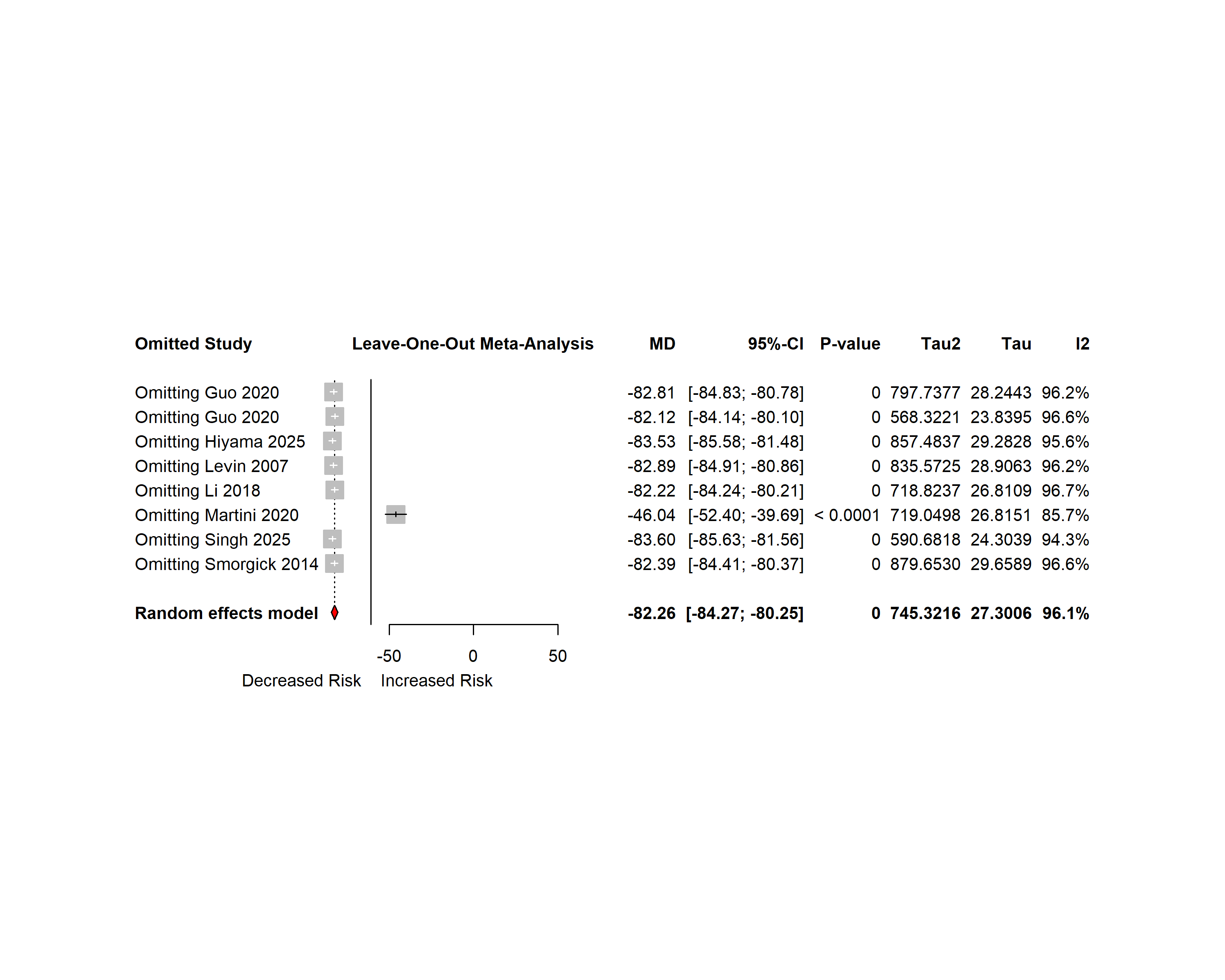

**Supplementary Figure 3:** Leave-one-out random-effects meta-analysis for operative time


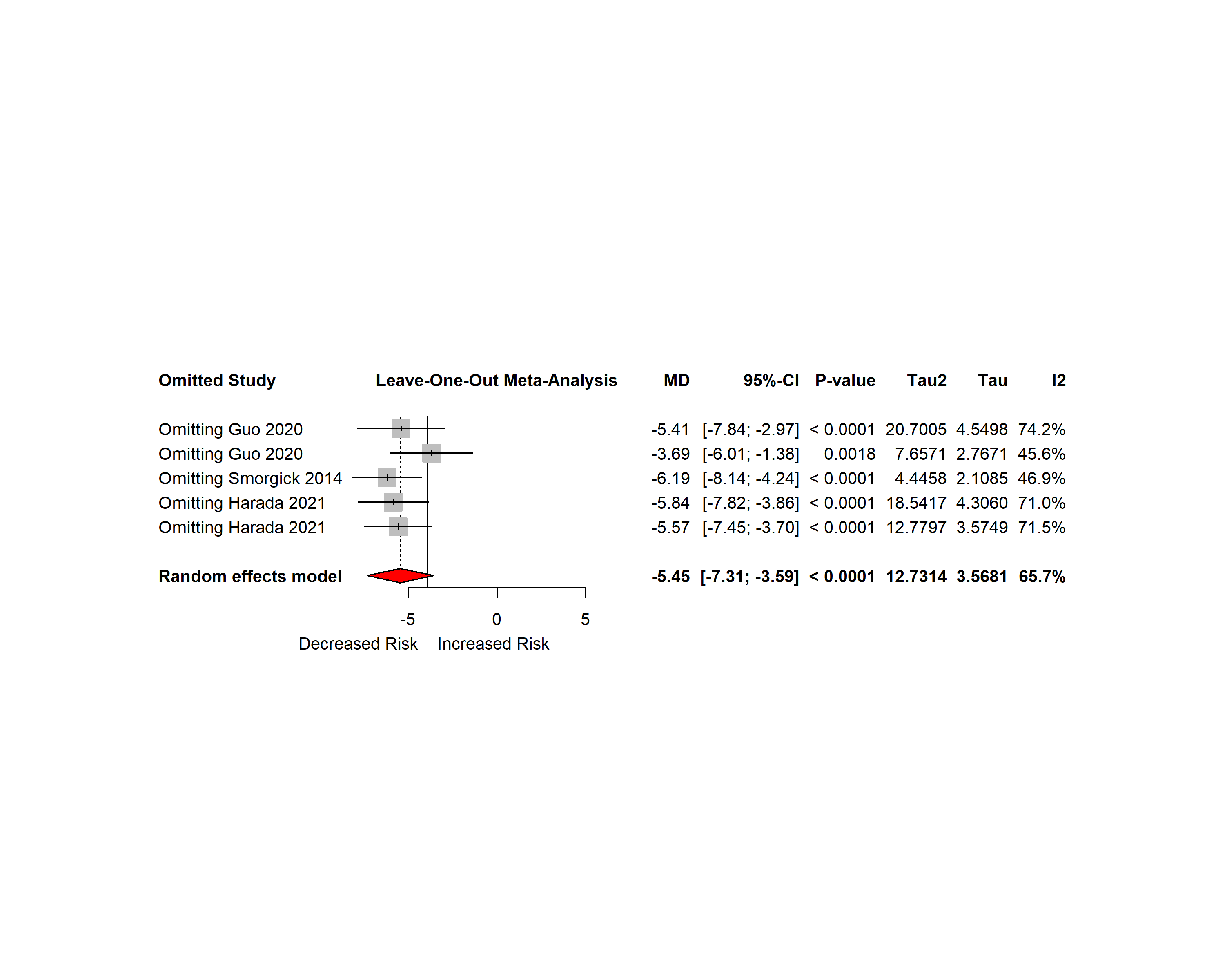

**Supplementary Figure 4:** Leave-one-out random-effects meta-analysis for Oswestry Disability Index at follow-up
